# Error Bounds for Discontinuous Finite Volume Discretisations of Brinkman Optimal Control Problems

**DOI:** 10.1007/s10915-018-0749-z

**Published:** 2018-06-09

**Authors:** S. Kumar, R. Ruiz-Baier, R. Sandilya

**Affiliations:** 1 Department of Mathematics, Indian Institute of Space Science and Technology, Trivandrum, 695547 India; 2 Mathematical Institute, Oxford University, A. Wiles Building, Radcliffe Observatory Quarter, Woodstock Road, Oxford, OX2 6GG UK; 3 Tata Institute of Fundamental Research, Centre For Applicable Mathematics, Bangalore, 560065 Karnataka India

**Keywords:** Optimal control problem, Brinkman equations, Variational control discretisation, Discontinuous finite volume methods, A priori error analysis, 49N05, 49K20, 65N30, 76D07, 76D55

## Abstract

We introduce a discontinuous finite volume method for the approximation of distributed optimal control problems governed by the Brinkman equations, where a force field is sought such that it produces a desired velocity profile. The discretisation of state and co-state variables follows a lowest-order scheme, whereas three different approaches are used for the control representation: a variational discretisation, and approximation through piecewise constant and piecewise linear elements. We employ the *optimise-then-discretise* approach, resulting in a non-symmetric discrete formulation. A priori error estimates for velocity, pressure, and control in natural norms are derived, and a set of numerical examples is presented to illustrate the performance of the method and to confirm the predicted accuracy of the generated approximations under various scenarios.

## Introduction

Fluid control problems are highly important in diverse fields of science and engineering. For example, they are encountered in the minimisation of drag, in the design of devices serving to increase mixing properties, in the reduction of turbulent kinetic energy, and in several other applications. Some of the earliest references to theoretical aspects of these control problems can be found in the classical works [1, 35]. The literature that relates to their numerical approximation is quite abundant, especially if associated to finite element methods (see e.g. [9, 26, 45, 47, 48] and the references therein). Works focusing on the approximation of control problems subject to Stokes and Navier–Stokes flows typically employ conforming discretisations for state, co-state and control variables. It has been found that the convergence rate of the control approximation is of $\mathcal {O}(h)$ and $\mathcal {O}(h^{\frac{3}{2}})$ for piecewise constant and piecewise linear discretisations, respectively. On the other hand, using the so-called variational discretisation approach (cf. [29], in which the control set is not discretised explicitly but recovered by a projection), it is possible to improve this convergence rate to $\mathcal {O}(h^2)$. Alternatively, a similar behaviour is observed if one uses graded meshes instead of uniform partitions [44], or if using piecewise constant control discretisations when state and adjoint variables are approximated with Lagrangian finite elements [42].

Error bounds for approximations to optimal control problems governed by flow equations are also available in the context of finite differences [25], spectral [17], mimetic [5], fully-mixed, discontinuous Galerkin [15, 21], and more recently, gradient discretisation methods [27].

The present paper focuses on finite volume element (FVE) approximations (methods where one introduces a dual mesh and reformulates a pure finite volume scheme in the form of a Petrov–Galerkin scheme). A priori error estimates for FVE schemes applied to linear elliptic and parabolic optimal control problems have been established in [38, 39]. These methods are based on the *optimise-then-discretise* approach, which we will adopt herein. In this context, we recall that the order in which the optimisation and discretisation steps are performed, results in different discrete adjoint equations and the solutions may not coincide (see the review [9] and the references therein). We will concentrate the analysis on a particular class of FVE schemes: a hybrid strategy called discontinuous finite volume (DFV) method, where discontinuous piecewise linear functions conform the trial space, and piecewise constant test functions are used in a FV fashion. The application of these schemes in the approximation of Stokes and related fluid problems can be found in e.g. [10–12, 24, 32, 33, 54].

Discontinuous approximations will be generally preferable to guarantee preservation of physically relevant properties. They would also be appropriate when the model exhibits rough coefficients and where sharp solutions are expected. Permeability fields possess this behaviour in many applicative scenarios, and DFV schemes would be of special interest. Other advantages of DFV formulations include flexibility for choosing accurate numerical fluxes, smaller dual control volumes, and suitability for error analysis in the $L^2$-norm. In the formulation advanced herein the momentum equation is tested against vector functions spanned by a basis associated to a dual grid, and the mass conservation equation is tested against piecewise constants defined on the primal mesh. Integration by parts on each dual element yields a classical finite volume scheme defined in terms of fluxes across the boundaries of dual elements. Then, some particular features of a given lumping map connecting discrete functions associated with the primal and dual meshes allow us to rewrite the formulation completely in terms of volume integrals involving primal elements, except for the zeroth-order term, the right-hand sides of the state and costate equations, and all jump terms that appear in the off-diagonal bilinear forms.

For non-viscous flow in porous media written in primal (pressure) formulation, the permeability tensor manifests itself as an anisotropic diffusion, and some methods are available for their successful discretisation. These include cell and vertex-centred schemes [23, 55], discrete duality finite volumes [16], high-order gradient reconstruction finite volume methods [40], mimetic schemes [18], anisotropic FVE methods [36], virtual finite volumes [22], or discontinuous immerse FVE schemes [37]. Even if the treatment is simpler in our case, as the inverse permeability is assumed isotropic, and it appears only in the drag term; our intention is not to perform a thorough comparison against these techniques, but rather to regard our contribution as a natural extension of the optimal control problems solved using specifically DFV methods (and having so far being constructed for systems governed by linear and semilinear elliptic, semilinear parabolic, and hyperbolic equations [49–52]) to the case of velocity control for the Brinkman equations. We emphasise once more that the discontinuous character of permeability fields represents a clear motivation for employing DFV methods. On the other hand, for the approximation of the control variable, we will discuss three alternatives: a variational discretisation approach, element-wise constant and element-wise linear discretisation.

The paper is structured in the following manner. The remainder of this section includes some standard notations, statement of the governing problem along with its weak formulation, and the corresponding optimality condition in continuous form. Next, in Sect. 2 we formulate the DFV scheme of the considered optimal control problem. Section 3 focuses on the development of a priori error estimates for different types of control discretisations. Finally, in Sect. 4 we summarise the solution algorithm and illustrate our theoretical error bounds and performance of the method by a set of numerical experiments.

Let $\varOmega \subset \mathbb {R}^d,\, d=2,3$, be a bounded convex polygonal domain with boundary $\partial \varOmega$. The outward unit normal vector to $\varOmega$ is denoted by $\varvec{n}$. Standard terminology will be employed for Sobolev spaces: $\mathbf {H}^1(\varOmega )=H^1(\varOmega )^d$ and $\mathbf {H}^1_0(\varOmega ):=\lbrace \varvec{v}\in \mathbf {H}^1(\varOmega ): \varvec{v}|_{\partial \varOmega }=\varvec{0}\rbrace$. The corresponding norms will be denoted by $\left\| \cdot \right\| _{1,\varOmega }$. We also consider the space of integrable functions with zero mean: $L_0^2(\varOmega )=\lbrace q \in L^2(\varOmega ): \int _{\varOmega } q\,\mathrm {d}\varvec{x}= 0 \rbrace$, and will write $\mathbf {L}^2(\varOmega )=L^2(\varOmega )^d$. By $\mathbf {div}$ we will denote the usual divergence operator $\,\mathrm{div}\,$ applied row-wise to a tensor, $\mathbf {I}$ will denote the $d\times d$ identity matrix, and $\varvec{0}$ will be used as a generic null vector.

**The Optimal Control Problem** Let us consider the following distributed optimal control problem

1.1 $$\begin{aligned} \min _{\varvec{u}\in \mathbf {U}_{\mathrm {ad}}} J(\varvec{u}) := \frac{1}{2} \Vert \varvec{y}-\varvec{y}_d\Vert _{0,\varOmega }^2 +\frac{\lambda }{2} \Vert \varvec{u}\Vert _{0,\varOmega }^2, \end{aligned}$$

governed by the Brinkman equations

1.2 $$\begin{aligned} \mathbf {K}^{-1}\varvec{y}-\mathbf {div}\,(\nu \varvec{\varepsilon }(\varvec{y})-p\mathbf {I})= & {} \varvec{u}+\varvec{f}\quad \text {in}\,\, \varOmega , \end{aligned}$$

1.3 $$\begin{aligned} \,\mathrm{div}\,\varvec{y}= & {} 0\quad \text {in} \,\, \varOmega , \end{aligned}$$

1.4 $$\begin{aligned} \varvec{y}= & {} \varvec{0}\quad \text {on} \,\, \partial \varOmega , \end{aligned}$$

where $\mathbf {U}_{\mathrm {ad}}$ is the set of feasible controls, defined for $-\infty \le a_j<b_j\le \infty ,\,\,j=1,\ldots ,d$ by

$$\begin{aligned} \mathbf {U}_{\mathrm {ad}}=\lbrace \varvec{u}\in \mathbf {L}^2(\varOmega ): a_j\le u_j \le b_j \quad \text {a.e. in } \varOmega \rbrace . \end{aligned}$$

This model describes the motion of an incompressible viscous fluid within an array of porous particles, and according to the flow regime characterised by the ratio between permeability and viscosity, it can represent both the Darcy and Stokes limits. Here $\varvec{y}$ denotes the fluid velocity, *p* is the pressure field, $\varvec{u}$ is the control variable, and $\lambda > 0$ is a given Tikhonov regularisation (or control cost) parameter. The quantity $\nu \varvec{\varepsilon }(\varvec{y})-p\mathbf {I}$ is the Cauchy (true stress) tensor, where $\varvec{\varepsilon }(\varvec{y})=\frac{1}{2}(\nabla \varvec{y}+\nabla \varvec{y}^T)$ is the infinitesimal rate of strain, $\nu (\varvec{x})$ is the dynamic viscosity of the fluid, and $\mathbf {K}(\varvec{x})$ stands for the permeability tensor of the medium divided by the viscosity. The forthcoming analysis requires that this matrix is isotropic. Here the desired velocity $\varvec{y}_d$ and the applied body force $\varvec{f}$ are known data with assumed regularity $\mathbf {L}^2(\varOmega )$ or $\mathbf {H}^1(\varOmega )$, depending on the specific case. One seeks to identify an additional source $\varvec{u}$ giving rise to a velocity $\varvec{y}$ in order to match a target velocity $\varvec{y}_d$. We stress that by proceeding analogously to the proof of [53, Theorem 2.37] it can be shown that the optimal value of the control $\varvec{u}$ is in $\mathbf {H}^1(\varOmega )$ under the assumption that $\varvec{y}_d\in \mathbf {L}^2(\varOmega )$. A similar observation has been made in [14], and used in the derivation of error estimates.

We assume that $\mathbf {K}$ is symmetric, uniformly bounded and positive definite, i.e., there exist two positive constants $k_1$ and $k_2$ such that

$$\begin{aligned} k_1|\xi |^2 \le \mathbf {K}(\varvec{x})\xi \cdot \xi \le k_2|\xi |^2, \quad \forall \xi \in \mathbb {R}^d,\varvec{x}\in \varOmega . \end{aligned}$$

We also assume that the variable viscosity satisfies

1.5 $$\begin{aligned} \exists \, \gamma _1, \nu _{\min }, \nu _{\max }>0: \, \forall s \in \mathbb {R}_+ ;\, \nu _{\min }<\nu (s)< \nu _{\max }, \, |\nu '(s)|\le \gamma _1, \end{aligned}$$

The weak formulation associated to the state equations (1.2)–(1.4) is given by: find $(\varvec{y},p)\in \mathbf {H}^1_0(\varOmega ) \times L^2_0(\varOmega )$ such that

1.6 $$\begin{aligned} \begin{aligned} a(\varvec{y},\varvec{v})+c(\varvec{y},\varvec{v})+b(\varvec{v},p)&=(\varvec{u}+\varvec{f},\varvec{v})_{0,\varOmega } \qquad \forall \varvec{v}\in \mathbf {H}^1_0(\varOmega ),\\ b(\varvec{y},q)&= 0 \qquad \forall q \in L^2_0(\varOmega ), \end{aligned} \end{aligned}$$

where the bilinear forms $a(\cdot ,\cdot ):\mathbf {H}^1_0(\varOmega )\times \mathbf {H}^1_0(\varOmega ) \rightarrow \mathbb {R},\,\, c(\cdot ,\cdot ):\mathbf {H}^1_0(\varOmega )\times \mathbf {H}^1_0(\varOmega ) \rightarrow \mathbb {R}$ and $b(\cdot ,\cdot ):\mathbf {H}^1_0(\varOmega ) \times L^2_0(\varOmega ) \rightarrow \mathbb {R}$ are defined as:

$$\begin{aligned} a(\varvec{y},\varvec{v}):=\int _{\varOmega } \mathbf {K}^{-1}\varvec{y}\cdot \varvec{v}\,\mathrm {d}\varvec{x}, \quad c(\varvec{y},\varvec{v}):=\int _{\varOmega }\nu \varvec{\varepsilon }(\varvec{y}):\varvec{\varepsilon }(\varvec{v})\,\mathrm {d}\varvec{x},\quad b(\varvec{v},q):=-\int _{\varOmega }q \,\mathrm{div}\,\varvec{v}\,\mathrm {d}\varvec{x}, \end{aligned}$$

for all $\varvec{y},\varvec{v}\in \mathbf {H}^1_0(\varOmega )$ and $q \in L^2_0(\varOmega )$. Above $(\cdot ,\cdot )_{0,\varOmega }$ stands for the scalar product in $\mathbf {L}^2(\varOmega )$ and $\left\| \cdot \right\| _{0,\varOmega }$ denotes the associated norm. The bilinear form $b(\cdot ,\cdot )$ relating the functional spaces for velocity and pressure satisfies the following Babuška-Brezzi condition (see [46], for example): there exists $\xi > 0$ such that

$$\begin{aligned} \inf _{q\in L^2_0(\varOmega )}\sup _{\varvec{0}\ne \varvec{v}\in \mathbf {H}^1_0(\varOmega )} \frac{b(\varvec{v},q)}{\left\| \varvec{v}\right\| _{1,\varOmega }\left\| q\right\| _{0,\varOmega }}\ge \xi , \end{aligned}$$

and, together with the ellipticity of $a(\cdot ,\cdot ) + c(\cdot ,\cdot )$, it implies the unique solvability of problem (1.6).

The optimal control problem (1.1)–(1.4) under consideration is strictly convex. Hence, it admits a unique optimal solution, and the first order necessary conditions are also sufficient for optimality (for details on well-posedness and first order optimality we refer to [35]). The optimality condition can be formulated as $J'(\varvec{u})(\tilde{\varvec{u}}-\varvec{u}) \ge 0 \quad \forall \tilde{\varvec{u}} \in \mathbf {U}_{\mathrm {ad}}$, and also rewritten as

1.7 $$\begin{aligned} (\varvec{w}+\lambda \varvec{u}, \tilde{\varvec{u}}-\varvec{u})_{0,\varOmega } \ge 0 \quad \forall \tilde{\varvec{u}} \in \mathbf {U}_{\mathrm {ad}}, \end{aligned}$$

where $\varvec{w}$ is the velocity associated with the adjoint equation

1.8 $$\begin{aligned} \mathbf {K}^{-1}\varvec{w}-\mathbf {div}(\nu \varvec{\varepsilon }(\varvec{w})+r\mathbf {I})= & {} \varvec{y}-\varvec{y}_d \quad \text {in } \varOmega , \end{aligned}$$

1.9 $$\begin{aligned} \,\mathrm{div}\,\varvec{w}= & {} 0\quad \text {in } \varOmega , \end{aligned}$$

1.10 $$\begin{aligned} \varvec{w}= & {} \varvec{0}\quad \text {on } \partial \varOmega . \end{aligned}$$

In turn, the variational inequality (1.7) can be equivalently recast in component-wise manner

$$\begin{aligned} u_j(\varvec{x})=P_{[a_j,b_j]}\left( \frac{-1}{\lambda } w_j(\varvec{x}) \right) \quad \text {a.e. in } \varOmega ,\,\, j=1,\ldots , d, \end{aligned}$$

where the operator *P* denotes a projection defined for a generic scalar function *g* as

$$\begin{aligned} P_{[a,b]}(g(\varvec{x}))=\max (a,\min (b,g(\varvec{x}))),\quad \text {a.e. in } \varOmega . \end{aligned}$$

It is not difficult to see that this projection satisfies the following regularity property (see also [43])

1.11 $$\begin{aligned} \left\| \nabla P_{[a,b]}(g)\right\| _{L^p(\varOmega )}\le \left\| \nabla g\right\| _{L^p(\varOmega )} \,\,\,\, \forall g \in W^{1,p}(\varOmega ),\,\,\,1\le p\le \infty . \end{aligned}$$

## Discontinuous Finite Volume Formulation

### Meshes, Discrete Spaces, and Interpolation Properties

Let us consider a regular, quasi-uniform partition $\mathcal {T}_h$ of $\bar{\varOmega }$ into closed triangles (or tetrahedra if $d=3$). By $h_T$ we denote the diameter of a given element $T\in \mathcal {T}_h$, and the global meshsize by $h=\max \limits _{T\in \mathcal {T}_h}h_T$. Moreover, let $\smash {\mathcal {E}_h}$ and $\mathcal {E}_h^\varGamma$ denote, respectively, the set of all faces and boundary faces in $\mathcal {T}_h$ (edges and boundary edges if $d=2$), and the symbol $h_e$ represents the length of the edge *e* (or the area of the face *e* if $d=3$). It follows from the definitions of $h_e$, $h_T$ and *h* that $h_{e}\le h_T^{d-1}\le h^{d-1}$.

**Fig. 1:**
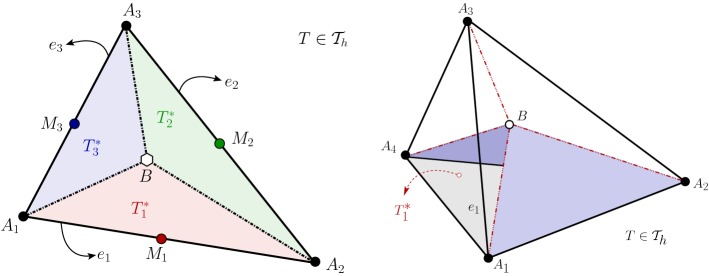
Left: sketch of a single primal element *T* in $\mathcal {T}_h$, and sub-elements $T_i^*$ belonging to the dual partition $\mathcal {T}_h^*$. Right: its three-dimensional counterpart, showing a tetrahedron *T* decomposed into four sub-tetrahedra

In addition to $\mathcal {T}_h$ (from now on, referred to as *primal mesh*), we introduce a dual partition in the following way. Each element $T\in \mathcal {T}_h$ is split into three sub triangles (or four sub-tetrahedra if $d=3$) $T^*_i$, $i=1,\ldots ,d+1$, by connecting the barycentre of the element to its corner nodes (see a schematic for $d=2$ and $d=3$ in Fig. 1). The set of all these elements generated by barycentric subdivison will be denoted by $\mathcal {T}_h^*$ and will be called the *dual partition* of $\mathcal {T}_h$. Let *e* be an interior face shared by two elements $T_1$ and $T_2$ in $\mathcal {T}_h$. By $\varvec{n}_1$ and $\varvec{n}_2$ we will denote unit normal vectors on *e* pointing outwards $T_1$ and $T_2$, respectively. The average ${\{\{\cdot \}\}_e}$ and jump $\llbracket \cdot \rrbracket _e$ operators defined on *e* for a generic scalar or vectorial field $\varvec{v}$ are:

$$\begin{aligned} \llbracket \varvec{v}\rrbracket _e:=\varvec{v}|_{{T_1}}-\varvec{v}|_{{T_2}},\quad \{\{\varvec{v}\}\}_e :=\frac{1}{2}\bigl (\varvec{v}|_{{T_1}}+\varvec{v}|_{{T_2}}\bigr ), \end{aligned}$$

respectively. If $e \in \mathcal {E}_h^\varGamma$, then we simply take $\{\{ \varvec{v}\}\}_e= \llbracket \varvec{v}\rrbracket _e = \varvec{v}$. Note that jump and averages are defined so that they preserve the dimension of the argument.

We denote by $P_m(T)$ the space of polynomials of degree less or equal than *m*, defined on $T\in \mathcal {T}_h$, and $\mathbf {P}_m(T)$ will denote its vectorial counterpart. A finite dimensional trial space (used for the state and co-state velocity approximation) associated with the primal partition $\mathcal {T}_h$ is

$$\begin{aligned} \mathbf {V}_h= \lbrace \varvec{v}_h \in \mathbf {L}^2(\varOmega ): \varvec{v}_h|_{T} \in \mathbf {P}_1(T),\ \forall T \in \mathcal {T}_h \rbrace , \end{aligned}$$

while the finite dimensional test space for velocity (and corresponding to the dual mesh $\mathcal {T}_h^*$) is

$$\begin{aligned} \mathbf {V}_h^*= \lbrace \varvec{v}_h \in \mathbf {L}^2(\varOmega ): \varvec{v}_h|_{T^*} \in \mathbf {P}_0(T^*),\ \forall T^* \in \mathcal {T}_h^* \rbrace . \end{aligned}$$

Moreover, the discrete space for state and co-state pressure approximation is defined as

$$\begin{aligned} Q_h = \lbrace q_h \in {L_0^2(\varOmega )}: q_h|_{T} \in {P_0(T)},\ \forall T \in \mathcal {T}_h \rbrace , \end{aligned}$$

and we define a space with higher regularity

$$\begin{aligned} \mathbf {V}(h)=\mathbf {V}_h+[\mathbf {H}^2(\varOmega )\cap \mathbf {H}^1_0(\varOmega )]. \end{aligned}$$

These spaces, associated with the two different meshes, are connected through the transfer operator $\gamma : \mathbf {V}(h)\rightarrow \mathbf {V}_h^*$, characterised in the following manner:

2.1 $$\begin{aligned} \gamma \varvec{v}|_{T^*}=\frac{1}{h_e}\int _{e}\varvec{v}|_{T^*}\,\mathrm {d}s, \quad \text {for }T^*\in \mathcal {T}_h^*. \end{aligned}$$

Some useful properties of this map are as follows.

#### Lemma 1

Let $\gamma$ be a transfer operator defined as in (2.1). Then

i) $\gamma$ is self-adjoint with respect to the $\mathbf {L}^2$-inner product, i.e.
  $$\begin{aligned} (\varvec{v}_h,\gamma \varvec{z}_h)_{0,\varOmega }=(\varvec{z}_h,\gamma \varvec{v}_h)_{0,\varOmega }, \quad \forall \varvec{v}_h, \varvec{z}_h\in \mathbf {V}_h. \end{aligned}$$
ii) The operator $\gamma$ is stable with respect to the norm $\left\| \cdot \right\| _{0,\varOmega }$, that is
  2.2 $$\begin{aligned} \left\| \gamma \varvec{v}_h\right\| _{0,\varOmega }=\left\| \varvec{v}_h\right\| _{0,\varOmega } \quad \forall \varvec{v}_h \in \mathbf {V}_h. \end{aligned}$$
  Moreover, if $\left| \left| \left| \varvec{v}_h\right| \right| \right| _{0,h}^2:=(\varvec{v}_h, \gamma \varvec{v}_h)_{0,\varOmega }$, then $\left| \left| \left| \cdot \right| \right| \right| _{0,h}$ and $\left\| \cdot \right\| _{0,\varOmega }$ are equivalent, with equivalence constants being independent of *h*.
iii) There exist constants $C_0$ and $C_1$ independent of *h* such that
  2.3 $$\begin{aligned} \left\| \varvec{v}_h-\gamma \varvec{v}_h\right\| _{0,T}&\le C_0 h_T \left\| \varvec{v}_h\right\| _{1,T} \;\;\;\;\forall \varvec{v}_h\in \mathbf {V}_h, \nonumber \\ (\varvec{v}_h,\gamma \varvec{v}_h)&\ge C_1\Vert \varvec{v}_h\Vert ^2_{0,\varOmega } \;\;\;\forall \varvec{v}_h\in \mathbf {V}_h \end{aligned}$$
iv) For all $\varvec{v}\in \mathbf {V}(h)$ and $T\in \mathcal {T}_h$, we have
  $$\begin{aligned} {\int _e (\varvec{v}-\gamma \varvec{v})\,\mathrm {d}s= \varvec{0}, \quad \int _e \llbracket \varvec{v}-\gamma \varvec{v}\rrbracket _e\,\mathrm {d}s= \varvec{0}, \quad \int _T (\varvec{v}-\gamma \varvec{v})\,\mathrm {d}\varvec{x}= \varvec{0}.} \end{aligned}$$

#### Proof

The proof of (*i*) and (*ii*) are given in [7] for the scalar version of the inter-mesh operator (2.1), and the same arguments can be used to extend their validity to the vector case. Property (*iii*) follows analogously to the proof of [20, Lemmas 2.3 and 2.6]. For a proof of (*iv*) we refer to [31]. $\square$

Let us stress that throughout the paper, the symbol *C* will represent a generic positive constant independent of meshsize *h*, that can take different values at different instances.

### Discrete Formulation for the State and Adjoint Equations

Let $\varvec{v}_h \in \mathbf {V}_h$. We proceed to test (1.2) and (1.3) against $\gamma \varvec{v}_h \in \mathbf {V}_h^*$ and $\phi _h\in Q_h$, respectively, and after integrating by parts the momentum equation on each dual element, and the mass conservation equation on each primal element, we end up with the following scheme: find $(\varvec{y}_h,p_h) \in \mathbf {V}_h \times Q_h$ such that

2.4 $$\begin{aligned} A_h(\varvec{y}_h,\varvec{v}_h)+c_h(\varvec{y}_h,\varvec{v}_h)+C_h(\varvec{v}_h,p_h)= & {} (\varvec{u}_h+\varvec{f},\gamma \varvec{v}_h)_{0,\varOmega }\quad \forall \varvec{v}_h\in \mathbf {V}_h, \end{aligned}$$

2.5 $$\begin{aligned} B_h(\varvec{y}_h,\phi _h)= & {} 0 \quad \forall \phi _h \in Q_h, \end{aligned}$$

where the discrete bilinear forms $A_h(\cdot ,\cdot )$, $B_h(\cdot ,\cdot )$, $c_h(\cdot ,\cdot )$ and $C_h(\cdot ,\cdot )$ are defined for all $\varvec{w}_h,\varvec{v}_h \in \mathbf {V}_h$ and $q_h \in Q_h$ as (see also [11]):

2.6 $$\begin{aligned} A_h(\varvec{w}_h,\varvec{v}_h):= & {} (\mathbf {K}^{-1}\varvec{w}_h, \gamma \varvec{v}_h)_{0,\varOmega },\quad B_h(\varvec{v}_h,q_h):= b(\varvec{v}_h,q_h)-\sum \limits _{e\in \smash {\mathcal {E}_h}}\int _e{\{\{q_h\varvec{n}\}\}_e}\cdot \llbracket \gamma \varvec{v}_h\rrbracket _e\,\mathrm {d}s, \nonumber \\ c_h(\varvec{w}_h,\varvec{v}_h):= & {} -\sum \limits _{T\in \mathcal {T}_h}\sum \limits _{j=1}^{d+1}\int _{A_{j+1}BA_j}\nu \varvec{\varepsilon }(\varvec{w}_h)\varvec{n}\cdot \gamma \varvec{v}_h \,\mathrm {d}s-\sum \limits _{e\in \smash {\mathcal {E}_h}}\int _e {\{\{ \nu \varvec{\varepsilon }(\varvec{w}_h)\varvec{n}\}\}_e}\cdot \llbracket \gamma \varvec{v}_h\rrbracket _e\,\mathrm {d}s\nonumber \\&\qquad -\sum \limits _{e\in \smash {\mathcal {E}_h}}\int _e {\{\{ \nu \varvec{\varepsilon }(\varvec{v}_h)\varvec{n}\}\}_e}\cdot \llbracket \gamma \varvec{w}_h\rrbracket _e\,\mathrm {d}s+\sum \limits _{e\in \smash {\mathcal {E}_h}}\int _e \frac{\alpha _d}{h_e^\delta }\llbracket \varvec{w}_h\rrbracket _e\cdot \llbracket \varvec{v}_h\rrbracket _e\,\mathrm {d}s, \nonumber \\ C_h(\varvec{v}_h,q_h):= & {} \sum \limits _{T\in \mathcal {T}_h}\sum \limits _{j=1}^{d+1}\int _{A_{j+1}BA_j} q_h\varvec{n}\cdot \gamma \varvec{v}_h\,\mathrm {d}s+\sum \limits _{e\in \smash {\mathcal {E}_h}}\int _e {\{\{ q_h\varvec{n}\}\}_e}\cdot \llbracket \gamma \varvec{v}_h\rrbracket _e\,\mathrm {d}s, \end{aligned}$$

where, $A_{d+2}=A_1$. With the help of Fig. 1 we see that, in 2D, for a fixed *j* the integrals $\int _{A_{j+1}BA_j}$ are considered over a path of two segments, whereas in 3D they are taken over a triangular facet. In any case, they contribute to construct the normal fluxes on the interior faces of each dual sub-element $T_j^*$, and so the symbol $\varvec{n}$ also denotes the outer normal on that sub-triangle or sub-tetrahedron. In addition, the constants $\alpha _d$ and $\delta =(d-1)^{-1}$ are parameters independent of *h* commonly used in interior penalty methods.

Proceeding analogously, we can write down a DFV formulation for the adjoint equation (1.8)–(1.10) as follows: find $(\varvec{w}_h,r_h) \in \mathbf {V}_h \times Q_h$ such that

$$\begin{aligned} A_h(\varvec{w}_h,\varvec{z}_h)+c_h(\varvec{w}_h,\varvec{z}_h)-C_h(\varvec{z}_h,r_h)= & {} (\varvec{y}_h-\varvec{y}_d,\gamma \varvec{z}_h)\quad \forall \varvec{z}_h\in \mathbf {V}_h, \\ B_h(\varvec{w}_h,\psi _h)= & {} 0 \quad \forall \psi _h \in Q_h. \end{aligned}$$

For the sake of our forthcoming analysis, we introduce the following discrete norms in $\mathbf {V}(h)$, which are naturally associated with the bilinear form $c_h(\cdot ,\cdot )$:

$$\begin{aligned} \left| \left| \left| \varvec{v}_h\right| \right| \right| _{1,h}^2:=\sum \limits _{T\in \mathcal {T}_h}|\varvec{v}_h|_{1,T}^2+\sum _{e\in \smash {\mathcal {E}_h}}h_e^{-\delta } \left\| \llbracket \varvec{v}_h\rrbracket _e\right\| _{0,e}^2, \quad \left| \left| \left| \varvec{v}_h\right| \right| \right| _{2,h}^2:=\left| \left| \left| \varvec{v}_h\right| \right| \right| _{1,h}^2+\sum _{T\in \mathcal {T}_h} h_T^2|\varvec{v}_h|_{2,T}^2, \end{aligned}$$

and we note that they are equivalent in $\mathbf {V}_h$. Moreover, we also have the following discrete Poincaré–Friedrichs inequality (see [11, pp. 457])

2.7 $$\begin{aligned} \left\| \varvec{v}_h\right\| _{0,\varOmega }\le C\left| \left| \left| \varvec{v}_h\right| \right| \right| _{2,h} \quad \forall \varvec{v}_h \in \mathbf {V}_h, \end{aligned}$$

and we can use Cauchy–Schwarz inequality and the definition of $\gamma$ to readily obtain

$$\begin{aligned} \frac{1}{h_e^{\delta /2}}\left\| \llbracket \gamma \varvec{v}_h\rrbracket _e\right\| _{0,e} \le \left( \frac{1}{h_e^{\delta }}\int _e \llbracket \varvec{v}_h\rrbracket _e^2 \,\mathrm {d}s\right) ^{1/2}. \end{aligned}$$

Proceeding analogously to [56, Lemma 6], we can establish the coercivity of the bilinear form $A_h(\cdot ,\cdot )$, stated in the following result.

#### Lemma 2

Let us assume that $\mathbf {K}^{-1} \in {\mathbf {W}}^{1,\infty }(T)$, for a generic element $T\in \mathcal {T}_h$. Then, for a sufficiently small meshsize *h*, there exists a constant $C > 0$ independent of *h*, such that

2.8 $$\begin{aligned} {A_h(\varvec{v}_h,\varvec{v}_h) \ge C \left\| \varvec{v}_h\right\| _{0,\varOmega }^2 \qquad \forall \varvec{v}_h \in \mathbf {V}_h.} \end{aligned}$$

#### Proof

Let $\mathbf {B}=\mathbf {K}^{-1}$ and consider its average tensor, defined on each primal element by

$$\begin{aligned} \overline{\mathbf {B}}:=\frac{1}{meas(T)}\int _T \mathbf {B}(\varvec{x}) \,\mathrm {d}\varvec{x}. \end{aligned}$$

Since $\mathbf {B}\in \mathbf {W}^{1,\infty } (T)$, we can infer that

2.9 $$\begin{aligned} \Vert \mathbf {B}-\overline{\mathbf {B}}\Vert _{[L^\infty (T)]^{d\times d}}\le C_3 h_T. \end{aligned}$$

Then, in view of Cauchy–Schwarz inequality together with properties (2.9) and (2.2), we can assert that

2.10 $$\begin{aligned} \int _T (\mathbf {B}-\overline{\mathbf {B}})\varvec{v}_h \cdot \gamma \varvec{v}_h \,\mathrm {d}\varvec{x}\le C_3 h \left\| \varvec{v}_h\right\| ^2_{0,T}. \end{aligned}$$

Now, let us write

$$\begin{aligned} \int _T\mathbf {B}\varvec{v}_h \cdot \gamma \varvec{v}_h \,\mathrm {d}\varvec{x}= \int _T \overline{\mathbf {B}}\varvec{v}_h \cdot \gamma \varvec{v}_h \,\mathrm {d}\varvec{x}-\int _T (\overline{\mathbf {B}}-\mathbf {B})\varvec{v}_h \cdot \gamma \varvec{v}_h \,\mathrm {d}\varvec{x}, \end{aligned}$$

and therefore, relations (2.10) and (2.3) lead to

$$\begin{aligned} (\mathbf {B}\varvec{v}_h, \gamma \varvec{v}_h)\ge C_1 \left\| \varvec{v}_h\right\| ^2_{0,\varOmega }-C_3 h \left\| \varvec{v}_h\right\| ^2_{0,\varOmega }\ge C \left\| \varvec{v}_h\right\| ^2_{0,\varOmega }, \end{aligned}$$

which holds provided *h* is sufficiently small, more precisely, if $$C_1-C_3h>0.$$$$\square $$

#### Lemma 3

The bilinear forms defined in (2.6) possess the following properties:

i) $A_h(\cdot ,\cdot )$ is continuous: there exists $C>0$, independent of *h*, such that
  $$\begin{aligned} |A_h(\varvec{v},\varvec{w})| \le C \left\| \varvec{v}\right\| _{0,\varOmega }\left\| \varvec{w}\right\| _{0,\varOmega } \qquad \forall \varvec{v},\varvec{w}\in \mathbf {V}(h), \end{aligned}$$
  and it satisfies the bound
  2.11 $$\begin{aligned} |A_h(\varvec{v}_h,\varvec{z}_h)-A_h(\varvec{z}_h,\varvec{v}_h)| \le Ch\left| \left| \left| \varvec{v}_h\right| \right| \right| _{2,h}\left| \left| \left| \varvec{z}_h\right| \right| \right| _{2,h} \quad \forall \varvec{v}_h,\varvec{z}_h\in \mathbf {V}_h. \end{aligned}$$
ii) For the non-symmetric bilinear form $c_h(\cdot ,\cdot )$ it holds that
  2.12 $$\begin{aligned} |c_h(\varvec{v},\varvec{w})|&\le C \left| \left| \left| \varvec{v}\right| \right| \right| _{2,h}\left| \left| \left| \varvec{w}\right| \right| \right| _{2,h}&\forall \varvec{v},\varvec{w}\in \mathbf {V}(h), \nonumber \\ c_h(\varvec{v}_h,\varvec{v}_h)&\ge C \left| \left| \left| \varvec{v}_h\right| \right| \right| _{2,h}^2&\forall \varvec{v}_h \in \mathbf {V}_h, \end{aligned}$$
  2.13 $$\begin{aligned} |c_h(\varvec{v}_h,\varvec{z}_h)-c_h(\varvec{z}_h,\varvec{v}_h)|&\le Ch\left| \left| \left| \varvec{v}_h\right| \right| \right| _{2,h}\left| \left| \left| \varvec{z}_h\right| \right| \right| _{2,h}&\forall \varvec{v}_h,\varvec{z}_h\in \mathbf {V}_h, \end{aligned}$$
  where for (2.12), $\alpha _d>0$ is assumed sufficiently large.
iii) The choice of approximation spaces $\mathbf {V}_h$ and $Q_h$ yields the condition
  2.14 $$\begin{aligned} \sup \limits _{\varvec{v}_h\in \mathbf {V}_h\setminus \{\varvec{0}\}} \frac{B_h(\varvec{v}_h,q_h)}{\left| \left| \left| \varvec{v}_h\right| \right| \right| _{2,h}} \ge \beta _1\left\| q_h\right\| _{0,\varOmega }, \end{aligned}$$
  where $\beta _1 >0$ is independent of *h*.
iv) The bilinear form $C_h(\cdot ,\cdot )$ satisfies
  2.15 $$\begin{aligned} |C_h(\varvec{v},q)|&{\le C \left| \left| \left| \varvec{v}\right| \right| \right| _{2,h}\left( \left\| q\right\| _{0,\varOmega }+ \left( \sum \limits _{T\in \mathcal {T}_h}h_T^2|q|^2_{1,T} \right) ^{1/2} \right) \quad \forall \varvec{v},\varvec{w}\in \mathbf {V}(h), \, q\in L_0^2(\varOmega )}, \nonumber \\ C_h(\varvec{v},q_h)&=-B_h(\varvec{v},q_h) \quad \forall \varvec{v}\in \mathbf {V}(h),\ q_h \in Q_h. \end{aligned}$$

#### Proof

For i) it suffices to apply the definition of $A_h(\cdot ,\cdot )$, together with relation (1.5), and the norm equivalence between $\left| \left| \left| \cdot \right| \right| \right| _{0,h}$ and $\left\| \cdot \right\| _{0,\varOmega }$. Results in ii) have been established in [33] and [7], whereas proofs for iii)-iv) can be found in [54]. $\square$

### Discretisation of the Control Variable

Let $\mathbf {U}_h \subseteq \mathbf {L}^2(\varOmega )$ denote the discrete control space, and let us introduce the discrete admissible space for the control field as $\mathbf {U}_{h,\mathrm {ad}}=\mathbf {U}_h \cap \mathbf {U}_{\mathrm {ad}}$. Three approaches are outlined in what follows.

**Variational Discretisation** In the so-called variational approach (cf. [29]), control variables are not discretised explicitly, that is, one simply takes $\mathbf {U}_h = \mathbf {L}^2(\varOmega )$ and in this case the discrete and continuous admissible spaces $\mathbf {U}_{h,\mathrm {ad}}$ and $\mathbf {U}_{\mathrm {ad}}$ coincide. Consequently, the control variable does not necessarily lie in a finite element space associated to $\mathcal {T}_h$, and typically one requires a nonstandard implementation and more involved stopping criteria for the algorithms of control computation. Discretisation errors using this method will be addressed in Sect. 3.2.

**Piecewise Linear Control Discretisation** Here we approximate the control variable with the similar elements as those employed for state and co-state velocity. That is,

$$\begin{aligned} \mathbf {U}^1_h=\lbrace \varvec{u}_h \in \mathbf {L}^2(\varOmega ): \varvec{u}_h|_T\in \mathbf {P}_1(T) \quad \forall T\in \mathcal {T}_h \rbrace . \end{aligned}$$

It is worthy to note that the state velocity space $\mathbf {V}_h$ coincides with the control space in the case of homogeneous Neumann boundary conditions, whereas for Dirichlet boundary data, we have $\mathbf {V}_h \subset \mathbf {U}^1_h$.

**Piecewise Constant Discretisation** In this case, the discrete control space is defined as

$$\begin{aligned} \mathbf {U}^0_h=\lbrace \varvec{u}_h \in \mathbf {L}^2(\varOmega ): \varvec{u}_h|_T\in \mathbf {P}_0(T)\quad \forall T\in \mathcal {T}_h \rbrace . \end{aligned}$$

The convergence properties associated with the above two approaches will be derived in Sect. 3.3, but already at this point we can apply Lemma 3 along with the Babuška–Brezzi theory for saddle point problems to ensure the unique solvability of (2.4)–(2.5), for a fixed control ${\varvec{u}}_h$.

Using relation (2.15), the DFV approximation of the continuous optimal system (1.1)–(1.4) can be summarised as: Find $(\varvec{y}_h,p_h,\varvec{w}_h,r_h,\varvec{u}_h)\in \mathbf {V}_h\times Q_h\times \mathbf {V}_h \times Q_h\times \mathbf {U}_{h,\mathrm {ad}}$ such that

2.16 $$\begin{aligned} A_h(\varvec{y}_h,\varvec{v}_h)+c_h(\varvec{y}_h,\varvec{v}_h)-B_h(\varvec{v}_h,p_h)&=(\varvec{u}_h+\varvec{f},\gamma \varvec{v}_h)_{0,\varOmega }&\forall \varvec{v}_h\in \mathbf {V}_h, \end{aligned}$$

2.17 $$\begin{aligned} B_h(\varvec{y}_h,\phi _h)&=0&\forall \phi _h \in Q_h, \end{aligned}$$

2.18 $$\begin{aligned} A_h(\varvec{w}_h,\varvec{z}_h)+c_h(\varvec{w}_h,\varvec{z}_h)+B_h(\varvec{z}_h,r_h)&=(\varvec{y}_h-\varvec{y}_d,\gamma \varvec{z}_h)_{0,\varOmega }&\forall \varvec{z}_h \in \mathbf {V}_h, \end{aligned}$$

2.19 $$\begin{aligned} B_h(\varvec{w}_h,\psi _h)&=0&\forall \psi _h \in Q_h, \end{aligned}$$

2.20 $$\begin{aligned} (\varvec{w}_h+\lambda \varvec{u}_h, \tilde{\varvec{u}}_h-\varvec{u}_h)_{0,\varOmega }&\ge 0&\forall \tilde{\varvec{u}}_h\in \mathbf {U}_{h,\mathrm {ad}}. \end{aligned}$$

## Convergence Analysis

In this section we provide a priori error estimates for DFV approximations of the state and adjoint equations, and for the three control discretisation approaches outlined in Sect. 2.3.

### Preliminaries

For a given control $\varvec{u}$ and $\varvec{f}$, let the pair $(\varvec{y}_h(\varvec{u}),p_h(\varvec{u}))$ be the solution of the following problem

3.1 $$\begin{aligned} A_h(\varvec{y}_h(\varvec{u}),\varvec{v}_h)+c_h(\varvec{y}_h(\varvec{u}),\varvec{v}_h)-B_h(\varvec{v}_h,p_h(\varvec{u}))= & {} (\varvec{u}+\varvec{f},\gamma \varvec{v}_h)_{0,\varOmega }\quad \forall \varvec{v}_h\in \mathbf {V}_h,\qquad \end{aligned}$$

3.2 $$\begin{aligned} B_h(\varvec{y}_h(\varvec{u}),\phi _h)= & {} 0 \quad \forall \phi _h \in Q_h. \end{aligned}$$

Similarly, for a given state velocity $\varvec{y}$, let $(\varvec{w}_h(\varvec{y}),r_h(\varvec{y}))$ be the solution of

3.3 $$\begin{aligned} A_h(\varvec{w}_h(\varvec{y}),\varvec{z}_h)+c_h(\varvec{w}_h(\varvec{y}),\varvec{z}_h)+B_h(\varvec{z}_h,r_h(\varvec{y}))= & {} (\varvec{y}-\varvec{y}_d,\gamma \varvec{z}_h)_{0,\varOmega }\quad \forall \varvec{z}_h\in \mathbf {V}_h,\qquad \end{aligned}$$

3.4 $$\begin{aligned} B_h(\varvec{w}_h(\varvec{y}),\psi _h)= & {} 0 \quad \forall \psi _h \in Q_h. \end{aligned}$$

We then proceed to decompose total errors in the following manner:

3.5 $$\begin{aligned} \varvec{y}-\varvec{y}_h=\varvec{y}-\varvec{y}_h(\varvec{u})+\varvec{y}_h(\varvec{u})-\varvec{y}_h, \quad \text {and}\quad \varvec{w}-\varvec{w}_h=\varvec{w}-\varvec{w}_h(\varvec{y})+\varvec{w}_h(\varvec{y})-\varvec{w}_h, \nonumber \\\end{aligned}$$

3.6 $$\begin{aligned} p-p_h=p-p_h(\varvec{u})+p_h(\varvec{u})-p_h, \quad \text {and}\quad r-r_h=r-r_h(\varvec{y})+r_h(\varvec{y})-r_h. \nonumber \\ \end{aligned}$$

Noting that $\varvec{y}_h=\varvec{y}_h(\varvec{u}_h)$, $p_h=p_h(\varvec{u}_h)$, $\varvec{w}_h=\varvec{w}_h(\varvec{y}_h)$, and $r_h=r_h(\varvec{y}_h)$, the following intermediate result is established.

#### Lemma 4

There exists a positive constant *C* independent of *h* such that the following estimates hold

3.7 $$\begin{aligned} \left| \left| \left| \varvec{y}_h(\varvec{u})-\varvec{y}_h\right| \right| \right| _{2,h}+\left\| p_h(\varvec{u})-p_h\right\| _{0,\varOmega }\le & {} C \left\| \varvec{u}-\varvec{u}_h\right\| _{0,\varOmega }, \end{aligned}$$

3.8 $$\begin{aligned} \left| \left| \left| \varvec{w}_h(\varvec{y})-\varvec{w}_h\right| \right| \right| _{2,h}+\left\| r_h(\varvec{y})-r_h\right\| _{0,\varOmega }\le & {} C \left\| \varvec{y}-\varvec{y}_h\right\| _{0,\varOmega }. \end{aligned}$$

#### Proof

Subtracting Eqs. (2.16) and (2.17) from (3.1) and (3.2), respectively, we have that the following relations hold for all $\varvec{v}_h \in \mathbf {V}_h$ and $\phi _h \in Q_h$

3.9 $$\begin{aligned} A_h(\varvec{y}_h(\varvec{u})-\varvec{y}_h,\varvec{v}_h)+c_h(\varvec{y}_h(\varvec{u})-\varvec{y}_h,\varvec{v}_h)-B_h(\varvec{v}_h,p_h(\varvec{u})-p_h)&=(\varvec{u}-\varvec{u}_h,\gamma \varvec{v}_h)_{0,\varOmega }, \end{aligned}$$

3.10 $$\begin{aligned} B_h(\varvec{y}_h(\varvec{u})-\varvec{y}_h,\phi _h)&=0. \end{aligned}$$

Adding (3.9) and (3.10) after choosing $\varvec{v}_h = \varvec{y}_h(\varvec{u})-\varvec{y}_h$ and $\phi _h=p_h(\varvec{u})-p_h$, implies that

$$\begin{aligned}&A_h(\varvec{y}_h(\varvec{u})-\varvec{y}_h,\varvec{y}_h(\varvec{u})-\varvec{y}_h)+c_h(\varvec{y}_h(\varvec{u})-\varvec{y}_h,\varvec{y}_h(\varvec{u})-\varvec{y}_h)\\&\quad =(\varvec{u}-\varvec{u}_h,\gamma (\varvec{y}_h(\varvec{u})-\varvec{y}_h))_{0,\varOmega }. \end{aligned}$$

In turn, using the coercivity of $A_h(\cdot ,\cdot )$ and $c_h(\cdot ,\cdot )$ in combination with (2.2) and (2.7), we obtain

$$\begin{aligned} \left\| \varvec{y}_h(\varvec{u})-\varvec{y}_h\right\| _{0,\varOmega }^2+\left| \left| \left| \varvec{y}_h(\varvec{u})-\varvec{y}_h\right| \right| \right| _{2,h}^2&\le C\bigl (\varvec{u}-\varvec{u}_h,\gamma (\varvec{y}_h(\varvec{u})-\varvec{y}_h)\bigr )_{0,\varOmega },\\&\le C\left\| \varvec{u}-\varvec{u}_h\right\| _{0,\varOmega }\left| \left| \left| \varvec{y}_h(\varvec{u})-\varvec{y}_h\right| \right| \right| _{2,h}, \end{aligned}$$

which readily yields the bound

3.11 $$\begin{aligned} \left| \left| \left| \varvec{y}_h(\varvec{u})-\varvec{y}_h\right| \right| \right| _{2,h} \le C\left\| \varvec{u}-\varvec{u}_h\right\| _{0,\varOmega }. \end{aligned}$$

On the other hand, applying the inf-sup condition (2.14), using (3.9), the boundedness of $A_h(\cdot ,\cdot )$ and $c_h(\cdot ,\cdot )$, along with (3.11), we realise that

3.12 $$\begin{aligned}&\left\| p_h-p_h(\varvec{u})\right\| _{0,\varOmega } \le \frac{1}{\beta _1}\sup \limits _{\varvec{v}_h\in \mathbf {V}_h\setminus \{\varvec{0}\}}\dfrac{B_h(v_h,p_h-p_h(\varvec{u}))}{\left| \left| \left| \varvec{v}_h\right| \right| \right| _{2,h}},\nonumber \\&\quad = \frac{1}{\beta _1}\sup _{\varvec{v}_h\in \mathbf {V}_h\setminus \{\varvec{0}\}}\dfrac{A_h(\varvec{y}_h(\varvec{u})-\varvec{y}_h,\varvec{v}_h)+c_h(\varvec{y}_h(\varvec{u})-\varvec{y}_h,\varvec{v}_h)+(\varvec{u}_h-\varvec{u},\gamma \varvec{v}_h)_{0,\varOmega }}{\left| \left| \left| \varvec{v}_h\right| \right| \right| _{2,h}}\nonumber \\&\quad \le C\left\| \varvec{u}-\varvec{u}_h\right\| _{0,\varOmega }. \end{aligned}$$

Relations (3.11) and (3.12) imply, in particular, that (3.7) holds. Next, we subtract Eqs. (2.18) and (2.19) from (3.3) and (3.4), respectively, and test the result against $\varvec{z}_h = \varvec{w}_h(\varvec{y})-\varvec{w}_h$ and $\psi _h=r_h(\varvec{y})-r_h$, which yields (3.8) after repeating the same steps as above. $\square$

#### Lemma 5

Assume that $\nu \in W^{2,\infty }(\varOmega )$ and that $\varvec{u},\varvec{f},\varvec{y}_d\in \mathbf {H}^1(\varOmega )$. Then, there exists a positive constant *C*, independent of *h*, such that

3.13 $$\begin{aligned} {\left\{ \begin{array}{ll} \left| \left| \left| \varvec{y}-\varvec{y}_h(\varvec{u})\right| \right| \right| _{2,h}+\left\| p-p_h(u)\right\| _{0,\varOmega } \le Ch\left( \left\| \varvec{y}\right\| _{2,\varOmega }+\left\| p\right\| _{1,\varOmega }\right) ,\\ \left| \left| \left| \varvec{w}-\varvec{w}_h(\varvec{y})\right| \right| \right| _{2,h}+\left\| r-r_h(y)\right\| \le Ch\left( \left\| \varvec{w}\right\| _{2,\varOmega }+\left\| r\right\| _{1,\varOmega }\right) , \end{array}\right. } \end{aligned}$$

3.14 $$\begin{aligned} {\left\{ \begin{array}{ll} \left\| \varvec{y}-\varvec{y}_h(\varvec{u})\right\| _{0,\varOmega } \le Ch^2 \left[ \left\| \varvec{y}\right\| _{2,\varOmega }+\left\| p\right\| _{1,\varOmega } +\left\| \varvec{u}\right\| _{1,\varOmega } +\left\| \varvec{f}\right\| _{1,\varOmega } \right] ,\\ \left\| \varvec{w}-\varvec{w}_h(\varvec{y})\right\| _{0,\varOmega } \le Ch^2 \left[ \left\| \varvec{w}\right\| _{2,\varOmega }+\left\| r\right\| _{1,\varOmega }+ \left\| \varvec{y}\right\| _{1,\varOmega } +\left\| \varvec{y}_d\right\| _{1,\varOmega }\right] . \end{array}\right. } \end{aligned}$$

#### Proof

We proceed analogously to the proof of [24, Theorem 3.1] and directly apply Lemma 3 to readily derive the following estimates:

$$\begin{aligned} \left| \left| \left| \varvec{y}-\varvec{y}_h(\varvec{u})\right| \right| \right| _{2,h}+\left\| p-p_h(u)\right\| _{0,\varOmega }\le & {} Ch\left( \left\| \varvec{y}\right\| _{2,\varOmega }+\left\| p\right\| _{1,\varOmega }\right) , \\ \left| \left| \left| \varvec{w}-\varvec{w}_h(\varvec{y})\right| \right| \right| _{2,h}+\left\| r-r_h(y)\right\| _{0,\varOmega }\le & {} Ch\left( \left\| \varvec{w}\right\| _{2,\varOmega }+\left\| r\right\| _{1,\varOmega }\right) . \end{aligned}$$

Next, the derivation of $\mathbf {L}^2$-estimates for $\varvec{y}-\varvec{y}_h(\varvec{u})$ and $\varvec{w}-\varvec{w}_h(\varvec{y})$ follows an Aubin-Nitsche duality argument. Let us consider the dual problem: find $(\varvec{z},\rho )\in \mathbf {H}^1_0(\varOmega )\times L^2_0(\varOmega )$ such that

3.15 $$\begin{aligned} \begin{aligned} \mathbf {K}^{-1}-\mathbf {div}(\nu \varvec{\varepsilon }(\varvec{z})-\rho \mathbf {I})&= \varvec{y}-\varvec{y}_h(\varvec{u}) \qquad \text {in }\varOmega , \\ \,\mathrm{div}\,\varvec{z}&= 0\qquad \text {in }\varOmega , \\ \varvec{z}&= 0 \qquad \text {on }\partial \varOmega , \end{aligned} \end{aligned}$$

which is uniquely solvable, and moreover the following $\mathbf {H}^2(\varOmega )\times H^1(\varOmega )$-regularity is satisfied:

3.16 $$\begin{aligned} \left\| \varvec{z}\right\| _{2,\varOmega }+\left\| \rho \right\| _{1,\varOmega } \le \left\| \varvec{y}-\varvec{y}_h(\varvec{u})\right\| _{0,\varOmega }. \end{aligned}$$

Let us denote by $\varvec{z}_I \in \mathbf {V}_h$ the usual continuous piecewise linear interpolant of $\varvec{z}$, satisfying the following approximation properties:

3.17 $$\begin{aligned} \left| \left| \left| \varvec{z}-\varvec{z}_I\right| \right| \right| _{2,h} \le Ch\left\| \varvec{z}\right\| _{2,\varOmega } \quad \text {and}\quad \left\| \varvec{z}-\varvec{z}_I\right\| _{0,\varOmega } \le Ch^2\left\| \varvec{z}\right\| _{2,\varOmega }. \end{aligned}$$

Also, let $\varPi _1$ denote the $L^2$-projection from $L^2_0(\varOmega )$ to $Q_h$, satisfying

$$\begin{aligned} \left\| \rho -\varPi _1 \rho \right\| _{0,\varOmega }\le Ch\left\| \rho \right\| _{1,\varOmega }. \end{aligned}$$

Multiplying (3.15) by $\varvec{y}-\varvec{y}_h(\varvec{u})$, integrating by parts, and using that $\llbracket \varvec{\varepsilon }(\varvec{z})\varvec{n}\rrbracket _e=\varvec{0}$ and $\llbracket \rho \rrbracket _e=0$, we can obtain

3.18 $$\begin{aligned} \left\| \varvec{y}-\varvec{y}_h(\varvec{u})\right\| _{0,\varOmega }^2= A_h^s(\varvec{y}-\varvec{y}_h(\varvec{u}),\varvec{z})+c_h^s(\varvec{y}-\varvec{y}_h(\varvec{u}),\varvec{z})-b_h^s(\varvec{y}-\varvec{y}_h(\varvec{u}),\rho ). \end{aligned}$$

where the auxiliary bilinear forms adopt the following expressions

$$\begin{aligned} A_h^s(\varvec{w}_h,\varvec{v}_h):= & {} (\mathbf {K}^{-1}\varvec{w}_h,\varvec{v}_h)_{0,\varOmega },\\ b_h^s(\varvec{v}_h,q_h):= & {} b(\varvec{v}_h,q_h)+\sum \limits _{e\in \smash {\mathcal {E}_h}}\int _e{\{\{ q_h\varvec{n}\}\}_e}\cdot \llbracket \varvec{v}_h\rrbracket _e\,\mathrm {d}s,\\ c_h^s(\varvec{w}_h,\varvec{v}_h):= & {} c(\varvec{w}_h,\varvec{v}_h)-\sum \limits _{e\in \smash {\mathcal {E}_h}}\int _e {\{\{ \nu \varvec{\varepsilon }(\varvec{w}_h)\varvec{n}\}\}_e}\cdot \llbracket \varvec{v}_h\rrbracket _e\,\mathrm {d}s\\&\qquad -\sum \limits _{e\in \smash {\mathcal {E}_h}}\int _e {\{\{ \nu \varvec{\varepsilon }(\varvec{v}_h)\varvec{n}\}\}_e}\cdot \llbracket \varvec{w}_h\rrbracket _e\,\mathrm {d}s+\sum \limits _{e\in \smash {\mathcal {E}_h}}\int _e \frac{\alpha _d}{h_e^\delta }\llbracket \varvec{w}_h\rrbracket _e\cdot \llbracket \varvec{v}_h\rrbracket _e\,\mathrm {d}s. \end{aligned}$$

Since $\varvec{z}_I \in \mathbf {V}_h$ is a continuous interpolant of $\varvec{z}$, we note that the pair $\bigl (\varvec{y}-\varvec{y}_h(\varvec{u}),p-p_h(\varvec{u})\bigr )$ will be a solution of the following problem

3.19 $$\begin{aligned} A_h(\varvec{y}-\varvec{y}_h(\varvec{u}),\varvec{z}_I)+c_h(\varvec{y}-\varvec{y}_h(\varvec{u}),\varvec{z}_I)+C_h(\varvec{z}_I,p-p_h(\varvec{u}))= & {} 0, \end{aligned}$$

3.20 $$\begin{aligned} B_h(\varvec{y}-\varvec{y}_h(\varvec{u}),\varPi _1 \rho )= & {} 0. \end{aligned}$$

Using the definition of $c_h(\cdot ,\cdot )$ and $C_h(\cdot ,\cdot )$ we can assert that

3.21 $$\begin{aligned} C_h(\varvec{z}_I,p-p_h(\varvec{u}))=-(\,\mathrm{div}\,\varvec{z}_I,p-p_h(\varvec{u}))_{\mathcal {T}_h}-(\nabla p,\varvec{z}_I- \gamma \varvec{z}_I)_{\mathcal {T}_h}, \end{aligned}$$

where the inner product over the primal mesh is understood as the sum of the inner products over each element in $\mathcal {T}_h$. On subtracting Eq. (3.19) from the sum of Eqs. (3.18) and (3.20), and using (3.21), it follows that

3.22 $$\begin{aligned}&\left\| \varvec{y}-\varvec{y}_h(\varvec{u})\right\| _{0,\varOmega }^2=\underbrace{\left[ A_h^s(\varvec{y}-\varvec{y}_h(\varvec{u}),\varvec{z})-A_h(\varvec{y}-\varvec{y}_h(\varvec{u}),\varvec{z}_I)\right] }_{R_1} + \underbrace{c_h^s(\varvec{y}-\varvec{y}_h(\varvec{u}),\varvec{z}-\varvec{z}_I)}_{R_2}\nonumber \\&\quad +\underbrace{\left[ c_h^s(\varvec{y}-\varvec{y}_h(\varvec{u}),\varvec{z}_I)-c_h(\varvec{y}-\varvec{y}_h(\varvec{u}),\varvec{z}_I)+\sum \limits _{T\in \mathcal {T}_h}\int _T (\varvec{z}_I- \gamma \varvec{z}_I)\cdot \nabla p\,\mathrm {d}\varvec{x}\right] }_{R_3}\nonumber \\&\quad +\underbrace{(p-p_h(\varvec{u}),\,\mathrm{div}\,\varvec{z}_I)_{0,\varOmega }}_{R_4}-\underbrace{b_h^s(\varvec{y}-\varvec{y}_h(\varvec{u}),\rho )+B_h(\varvec{y}-\varvec{y}_h(\varvec{u}),\varPi _1 \rho )}_{R_5}. \end{aligned}$$

Notice that the estimation of $R_1$ results as a combination of the boundedness of $\mathbf {K}^{-1}$, assumption (1.5), the bounds (3.17), the self-adjointness and approximation properties of $\gamma$ stated in (3.16), and Cauchy–Schwarz inequality. This gives

$$\begin{aligned} R_1\le & {} |(\varvec{y}-\varvec{y}_h(\varvec{u}),\mathbf {K}^{-1}\varvec{z})_{0,\varOmega }-(\mathbf {K}^{-1}(\varvec{y}-\varvec{y}_h(\varvec{u})),\gamma \varvec{z}_I)_{0,\varOmega }|\\\le & {} {C} |(\varvec{y}-\varvec{y}_h(\varvec{u}),\varvec{z}-\varvec{z}_I)_{0,\varOmega }+(\varvec{y}-\varvec{y}_h(\varvec{u})-\gamma (\varvec{y}-\varvec{y}_h(\varvec{u})), \varvec{z}_I)_{0,\varOmega }|\\\le & {} C(h^2\left\| \varvec{y}-\varvec{y}_h(\varvec{u})\right\| _{0,\varOmega }\left\| \varvec{z}\right\| _{2,\varOmega }+h\left| \left| \left| \varvec{y}-\varvec{y}_h(\varvec{u})\right| \right| \right| _{2,h}\left\| \varvec{z}_I\right\| _{0,\varOmega })\\\le & {} Ch^2(\left\| \varvec{y}-\varvec{y}_h(\varvec{u})\right\| _{0,\varOmega }^2+\left\| \varvec{y}\right\| _{2,\varOmega }\left\| \varvec{y}-\varvec{y}_h(\varvec{u})\right\| _{0,\varOmega }), \end{aligned}$$

where the last inequality follows from (3.13). For the second term we employ the definition of $c_h(\cdot ,\cdot )$, and relations (3.17),(3.16) to verify that

$$\begin{aligned} R_2 \le Ch^2\left\| \varvec{y}\right\| _{2,\varOmega }\left\| \varvec{z}\right\| _{2,\varOmega } \le Ch^2\left\| \varvec{y}\right\| _{2,\varOmega }\left\| \varvec{y}-\varvec{y}_h(\varvec{u})\right\| _{0,\varOmega }. \end{aligned}$$

Bounds for the remaining terms can be obtained following the proof of [33, Theorem 3.4] and [24, Theorem 3.2], as follows

$$\begin{aligned} R_3\le & {} Ch^2 \bigl [ \left\| \varvec{y}\right\| _{2,\varOmega }+\left\| \varvec{u}\right\| _{1,\varOmega } +\left\| \varvec{f}\right\| _{1,\varOmega } \bigr ]\left\| \varvec{y}-\varvec{y}_h(\varvec{u})\right\| _{0,\varOmega },\\ R_4\le & {} |(p-p_h(\varvec{u}),\,\mathrm{div}\,(\varvec{z}-\varvec{z}_I))_{0,\varOmega }| \le Ch^2\left\| p\right\| _{1,\varOmega }\left\| \varvec{y}-\varvec{y}_h(\varvec{u})\right\| _{0,\varOmega },\\ R_5\le & {} Ch^2\left\| \varvec{y}\right\| _{2,\varOmega }\left\| \varvec{y}-\varvec{y}_h(\varvec{u})\right\| _{0,\varOmega }. \end{aligned}$$

Combining the five estimates above with (3.22), we straightforwardly obtain

$$\begin{aligned} \left\| \varvec{y}-\varvec{y}_h(\varvec{u})\right\| _{0,\varOmega } \le Ch^2 \left[ \left\| \varvec{y}\right\| _{2,\varOmega }+\left\| p\right\| _{1,\varOmega } +\left\| \varvec{u}\right\| _{1,\varOmega } +\left\| \varvec{f}\right\| _{1,\varOmega } \right] , \end{aligned}$$

and very much in the same way, one arrives at

$$\begin{aligned} \left\| \varvec{w}-\varvec{w}_h(\varvec{y})\right\| _{0,\varOmega } \le Ch^2 \left[ \left\| \varvec{w}\right\| _{2,\varOmega }+\left\| r\right\| _{1,\varOmega }+ \left\| \varvec{y}\right\| _{1,\varOmega } +\left\| \varvec{y}_d\right\| _{1,\varOmega }\right] . \end{aligned}$$

$$\square $$

Now, for a given control $\varvec{u}$, let $(\varvec{w}_h(\varvec{u}),r_h(\varvec{u}))$ be the solution of

$$\begin{aligned} A_h(\varvec{w}_h(\varvec{u}),\varvec{z}_h)+c_h(\varvec{w}_h(\varvec{u}),\varvec{z}_h)+B_h(\varvec{z}_h,r_h(\varvec{u}))&= (\varvec{y}_h(\varvec{u})-\varvec{y}_d,\gamma \varvec{z}_h)_{0,\varOmega }&\forall \varvec{z}_h\in \mathbf {V}_h,\\ B_h(\varvec{w}_h(\varvec{u}),\psi _h)&=0&\forall \psi _h \in Q_h, \end{aligned}$$

and notice that similar arguments as those appearing in the proof of Lemma 5 and in the derivation of the estimate $\left\| \varvec{y}-\varvec{y}_h(\varvec{u})\right\| _{0,\varOmega } \le Ch^2$, will readily lead to

3.23 $$\begin{aligned} \left\| \varvec{w}-\varvec{w}_h(\varvec{u})\right\| _{0,\varOmega } \le Ch^2. \end{aligned}$$

The following result plays a vital role in deriving error estimates of the control, state and co-state variables. Its proof is similar to that in [38, Theorem 4.1].

#### Lemma 6

Assume that $\nu \in W^{2,\infty }(\varOmega )$ and $\varvec{u},\varvec{f}, \varvec{y}_d\in \mathbf {H}^1(\varOmega )$. Then

3.24 $$\begin{aligned} (\varvec{w}-\varvec{w}_h,\varvec{u}_h-\varvec{u})_{0,\varOmega }&\le Ch^2[ \left\| \varvec{y}\right\| _{2,\varOmega }+\left\| p\right\| _{1,\varOmega } +\left\| \varvec{u}\right\| _{1,\varOmega } +\left\| \varvec{f}\right\| _{1,\varOmega } +\left\| \varvec{w}\right\| _{2,\varOmega }+\left\| r\right\| _{1,\varOmega } \nonumber \\&\qquad + \left\| \varvec{y}\right\| _{1,\varOmega } +\left\| \varvec{y}_d\right\| _{1,\varOmega }]\left\| \varvec{u}_h-\varvec{u}\right\| _{0,\varOmega }+Ch\left\| \varvec{u}_h-\varvec{u}\right\| _{0,\varOmega }^2, \end{aligned}$$

where $C>0$ is independent of *h*.

#### Proof

We split $(\varvec{w}-\varvec{w}_h,\varvec{u}_h-\varvec{u})_{0,\varOmega }$ as

3.25 $$\begin{aligned} (\varvec{w}-\varvec{w}_h,\varvec{u}_h-\varvec{u})_{0,\varOmega }&=(\varvec{w}-\varvec{w}_h(y),\varvec{u}_h-\varvec{u})_{0,\varOmega }+(\varvec{w}_h(y)-\varvec{w}_h\nonumber \\&\quad \ -\gamma (\varvec{w}_h(y)-\varvec{w}_h),\varvec{u}_h-\varvec{u})_{0,\varOmega }\nonumber \\&\quad \ +(\gamma (\varvec{w}_h(y)-\varvec{w}_h),\varvec{u}_h-\varvec{u})_{0,\varOmega }. \end{aligned}$$

Then, using the approximation property of $\gamma$ together with Lemmas 4 and 5 implies

3.26 $$\begin{aligned}&(\varvec{w}-\varvec{w}_h(y),\varvec{u}_h-\varvec{u})_{0,\varOmega }+(\varvec{w}_h(y)-\varvec{w}_h-\gamma (\varvec{w}_h(y)-\varvec{w}_h),\varvec{u}_h-\varvec{u})_{0,\varOmega }\nonumber \\&\qquad \le \left\| \varvec{w}-\varvec{w}_h(y)\right\| _{0,\varOmega }\left\| \varvec{u}_h-\varvec{u}\right\| _{0,\varOmega }+Ch\left\| \varvec{y}-\varvec{y}_h\right\| _{0,\varOmega }\left\| \varvec{u}_h-\varvec{u}\right\| _{0,\varOmega }\nonumber \\&\qquad \le \left\| \varvec{w}-\varvec{w}_h(y)\right\| _{0,\varOmega }\left\| \varvec{u}_h-\varvec{u}\right\| _{0,\varOmega }+Ch(\left\| \varvec{y}-\varvec{y}_h(\varvec{u})\right\| _{0,\varOmega }+\left\| \varvec{u}_h-\varvec{u}\right\| _{0,\varOmega })\left\| \varvec{u}_h-\varvec{u}\right\| _{0,\varOmega }\nonumber \\&\qquad \le Ch^2 \left[ \left\| \varvec{w}\right\| _{2,\varOmega }+\left\| r\right\| _{1,\varOmega }+ \left\| \varvec{y}\right\| _{1,\varOmega } +\left\| \varvec{y}_d\right\| _{1,\varOmega }\right] \left\| \varvec{u}_h-\varvec{u}\right\| _{0,\varOmega }\nonumber \\&\qquad \qquad +Ch(h^2 \left[ \left\| \varvec{y}\right\| _{2,\varOmega }+\left\| p\right\| _{1,\varOmega } +\left\| \varvec{u}\right\| _{1,\varOmega } +\left\| \varvec{f}\right\| _{1,\varOmega } \right] +\left\| \varvec{u}_h-\varvec{u}\right\| _{0,\varOmega })\left\| \varvec{u}_h-\varvec{u}\right\| _{0,\varOmega }\nonumber \\&\qquad \le Ch^2 \left[ \left\| \varvec{w}\right\| _{2,\varOmega }+\left\| r\right\| _{1,\varOmega }+ \left\| \varvec{y}\right\| _{1,\varOmega } +\left\| \varvec{y}_d\right\| _{1,\varOmega }\right] \left\| \varvec{u}_h-\varvec{u}\right\| _{0,\varOmega }+Ch\left\| \varvec{u}_h-\varvec{u}\right\| _{0,\varOmega }^2. \end{aligned}$$

Now we subtract (3.1) and (3.2) from (2.16) and (2.17), respectively and test the result against $\varvec{v}_h = \varvec{w}_h(\varvec{y})-\varvec{w}_h$ and $\phi _h=r_h(\varvec{y})-r_h$ to obtain the relation

3.27 $$\begin{aligned} (\gamma (\varvec{w}_h(\varvec{y})-\varvec{w}_h),\varvec{u}_h-\varvec{u})_{0,\varOmega }=&\, A_h(\varvec{y}_h-\varvec{y}_h(\varvec{u}),\varvec{w}_h(\varvec{y})-\varvec{w}_h)\nonumber \\&+c_h(\varvec{y}_h-\varvec{y}_h(\varvec{u}),\varvec{w}_h(\varvec{y})-\varvec{w}_h)\nonumber \\&-B_h(\varvec{w}_h(\varvec{y})-\varvec{w}_h,p_h-p_h(\varvec{u}))\nonumber \\&+B(\varvec{y}_h-\varvec{y}_h(\varvec{u}),r_h(\varvec{y})-r_h). \end{aligned}$$

Similarly, subtracting Eqs. (2.18) and (2.19) from (3.3) and (3.4), respectively, and taking $\varvec{z}_h = \varvec{y}_h-\varvec{y}_h(\varvec{u})$ and $\psi _h=p_h-p_h(\varvec{u})$, we can assert that

3.28 $$\begin{aligned}&A_h(\varvec{w}_h(\varvec{y})-\varvec{w}_h,\varvec{y}_h-\varvec{y}_h(\varvec{u}))+c_h(\varvec{w}_h(\varvec{y})-\varvec{w}_h,\varvec{y}_h-\varvec{y}_h(\varvec{u}))\nonumber \\&\quad =(\varvec{y}-\varvec{y}_h,\gamma (\varvec{y}_h-\varvec{y}_h(\varvec{u})))_{0,\varOmega }-B_h(\varvec{y}_h-\varvec{y}_h(\varvec{u}),r_h(\varvec{y})-r_h)\nonumber \\&\qquad +B(\varvec{w}_h(\varvec{y})-\varvec{w}_h,p_h-p_h(\varvec{u})). \end{aligned}$$

Adding (3.27) and (3.28) and using that $(\varvec{y}_h-\varvec{y}_h(\varvec{u}),\gamma (\varvec{y}_h-\varvec{y}_h(\varvec{u})))_{0,\varOmega }\ge 0$, we arrive at

$$\begin{aligned}&(\gamma (\varvec{w}_h(\varvec{y})-\varvec{w}_h),\varvec{u}_h-\varvec{u})_{0,\varOmega }\\&\ \le [A_h(\varvec{y}_h-\varvec{y}_h(\varvec{u}),\varvec{w}_h(\varvec{y})-\varvec{w}_h)-A_h(\varvec{w}_h(\varvec{y})-\varvec{w}_h,\varvec{y}_h-\varvec{y}_h(\varvec{u}))]\\&\quad \ \, +[c_h(\varvec{y}_h-\varvec{y}_h(\varvec{u}),\varvec{w}_h(\varvec{y})-\varvec{w}_h)-c_h(\varvec{w}_h(\varvec{y})-\varvec{w}_h,\varvec{y}_h-\varvec{y}_h(\varvec{u}))]\\&\quad +(\varvec{y}-\varvec{y}_h(\varvec{u}),\gamma (\varvec{y}_h-\varvec{y}_h(\varvec{u})))_{0,\varOmega }\\&\ \le Ch\left| \left| \left| \varvec{y}_h-\varvec{y}_h(\varvec{u})\right| \right| \right| _{2,h}\left| \left| \left| \varvec{w}_h(\varvec{y})-\varvec{w}_h\right| \right| \right| _{2,h}+\left\| \varvec{y}-\varvec{y}_h(\varvec{u})\right\| _{0,\varOmega }\left| \left| \left| \varvec{y}_h-\varvec{y}_h(\varvec{u})\right| \right| \right| _{2,h}, \end{aligned}$$

where we have used relations (2.2), (2.7), (2.11) and (2.13). An application of Lemmas 4 and 5 in the above inequality leads to the following bound

3.29 $$\begin{aligned} (\gamma (\varvec{w}_h(\varvec{y})-\varvec{w}_h),\varvec{u}_h-\varvec{u})_{0,\varOmega } \le&\, Ch^2\left[ \left\| \varvec{y}\right\| _{2,\varOmega }+\left\| p\right\| _{1,\varOmega } \right. \nonumber \\&\left. +\left\| \varvec{u}\right\| _{1,\varOmega } +\left\| \varvec{f}\right\| _{1,\varOmega } \right] \left\| \varvec{u}_h-\varvec{u}\right\| _{0,\varOmega }\nonumber \\&+ Ch\left\| \varvec{u}_h-\varvec{u}\right\| _{0,\varOmega }^2. \end{aligned}$$

Finally, inserting estimates (3.26) and (3.29) into (3.25), we get the required result. $\square$

#### Remark 1

(Right-hand side regularity) According to the contributions [8, 19, 28, 30] (see also the references therein), for linear finite volume element methods applied to second order elliptic problems, the optimal error estimates (establishing second order accuracy in the $L^2-$ norm) can be achieved under the assumption that the source term is in $H^1(\varOmega )$. However, assuming that the right-hand side is in $H^1(\varOmega )$ does not imply that the exact solution is in $H^3(\varOmega )$, as discussed in e.g. [19]. Some counterexamples are actually given in [28, 30] to confirm that the optimal $L^2-$ error estimates cannot be derived if one only assumes that the forcing term is in $L^2(\varOmega )$. Proceeding analogously to the analysis of standard finite volume methods, optimal error estimates in the $L^2-$ norm have been derived by taking the source term in $H^1(\varOmega )$ (see for instance [19, 24] and their references, for the specific case of DFV methods applied to elliptic and Stokes problems). Following the analysis of [31], one can derive the error estimates given in Lemmas 5 and 6 under the less restrictive assumption that $\varvec{f}$ and $\varvec{y}_d$ are in $\mathbf {H}^1(T)$, that is, locally-$\mathbf {H}^1$.

### Error Estimates Under Variational Discretisation

#### Theorem 1

Let $(\varvec{y}_h,\varvec{w}_h)$ be DFV approximations of $(\varvec{y},\varvec{w})$ and let $\varvec{u}_h$ denote a variational discretisation of $\varvec{u}$. Then there exists a positive constant *C* independent of *h*, but depending on $\lambda$, such that the following estimates hold:

3.30 $$\begin{aligned} \left\| \varvec{u}-\varvec{u}_h\right\| _{0,\varOmega }&\le Ch^2[ \left\| \varvec{y}\right\| _{2,\varOmega }+\left\| p\right\| _{1,\varOmega } +\left\| \varvec{u}\right\| _{1,\varOmega } +\left\| \varvec{f}\right\| _{1,\varOmega } +\left\| \varvec{w}\right\| _{2,\varOmega }+\left\| r\right\| _{1,\varOmega } \nonumber \\&\qquad + \left\| \varvec{y}\right\| _{1,\varOmega } +\left\| \varvec{y}_d\right\| _{1,\varOmega }], \end{aligned}$$

3.31 $$\begin{aligned} \left\| \varvec{y}-\varvec{y}_h\right\| _{0,\varOmega }&\le Ch^2[ \left\| \varvec{y}\right\| _{2,\varOmega }+\left\| p\right\| _{1,\varOmega } +\left\| \varvec{u}\right\| _{1,\varOmega } +\left\| \varvec{f}\right\| _{1,\varOmega } +\left\| \varvec{w}\right\| _{2,\varOmega }+\left\| r\right\| _{1,\varOmega } \nonumber \\&\qquad + \left\| \varvec{y}\right\| _{1,\varOmega } +\left\| \varvec{y}_d\right\| _{1,\varOmega }], \end{aligned}$$

3.32 $$\begin{aligned} \left\| \varvec{w}-\varvec{w}_h\right\| _{0,\varOmega }&\le Ch^2[ \left\| \varvec{y}\right\| _{2,\varOmega }+\left\| p\right\| _{1,\varOmega } +\left\| \varvec{u}\right\| _{1,\varOmega } +\left\| \varvec{f}\right\| _{1,\varOmega } +\left\| \varvec{w}\right\| _{2,\varOmega }+\left\| r\right\| _{1,\varOmega } \nonumber \\&\qquad + \left\| \varvec{y}\right\| _{1,\varOmega } +\left\| \varvec{y}_d\right\| _{1,\varOmega }]. \end{aligned}$$

#### Proof

We recall the continuous variational inequality

3.33 $$\begin{aligned} {(\varvec{w}+\lambda \varvec{u}, \tilde{\varvec{u}}-\varvec{u})}_{0,\varOmega } \ge 0 \quad \forall \tilde{\varvec{u}}\in \mathbf {U}_{\mathrm {ad}}, \end{aligned}$$

and the discrete variational inequality under variational discretisation

3.34 $$\begin{aligned} (\varvec{w}_h+\lambda \varvec{u}_h, \tilde{\varvec{u}}_h-\varvec{u}_h)_{0,\varOmega } \ge 0 \quad \forall \tilde{\varvec{u}}_h\in \mathbf {U}_{\mathrm {ad}}. \end{aligned}$$

Choosing $\tilde{\varvec{u}}=\varvec{u}_h$ and $\tilde{\varvec{u}}_h=\varvec{u}$ in (3.33) and (3.34), respectively, and adding up the resulting inequalities, yields

$$\begin{aligned} (\varvec{w}+\lambda \varvec{u}, \varvec{u}_h-\varvec{u})_{0,\varOmega }+(\varvec{w}_h+\lambda \varvec{u}_h, \varvec{u}-\varvec{u}_h)_{0,\varOmega } \ge 0, \end{aligned}$$

and rearranging terms, we get

3.35 $$\begin{aligned} \lambda \left\| \varvec{u}-\varvec{u}_h\right\| _{0,\varOmega }^2 \le (\varvec{w}-\varvec{w}_h ,\varvec{u}_h-\varvec{u})_{0,\varOmega }. \end{aligned}$$

An application of (3.24) in (3.35) implies the required result (3.30). Using (3.5) and the triangle inequality together with Lemmas 4 and 5, and result (3.30), the remaining estimates (3.31)–(3.32) follow in a straightforward manner. $\square$

### $\mathbf {L}^2$-Error Estimates for Fully Discretised Controls

A discrete admissible control $\tilde{\varvec{u}}_h=(\tilde{u}_{h,j})_{j=1}^d \in \mathbf {U}_{h,\mathrm {ad}}$ is defined component-wise and locally as

3.36 $$\begin{aligned} \tilde{u}_{h,j}= {\left\{ \begin{array}{ll} a_j &{} \text {if } \min \limits _{x\in T} u_j(\varvec{x}) = a_j,\\ b_j &{} \text {if } \max \limits _{x\in T} u_j(\varvec{x}) = b_j,\\ \tilde{I}_h u_j &{} \text {otherwise}, \end{array}\right. } \end{aligned}$$

where $\tilde{I}_h u_j$ is the Lagrange interpolant of $u_j$. To avoid ambiguity, we choose *h* sufficiently small so that $\min _{x\in T} u_j(\varvec{x}) = a_j$ and $\max _{x\in T} u_j(\varvec{x}) = b_j$ do not occur simultaneously within the same element $T\in \mathcal {T}_h$. Next, we proceed to group the elements in the primal mesh into three categories: $\mathcal {T}_h= \mathcal {T}_{h,1}^j \cup \mathcal {T}_{h,2}^j \cup \mathcal {T}_{h,3}^j$ with $\mathcal {T}_{h,m}^j \cap \mathcal {T}_{h,n}^j=\emptyset$ for $m \ne n$ according to the value of $u_j(\varvec{x})$ on *T*. These sets are defined as

$$\begin{aligned} \mathcal {T}_{h,1}^j= & {} \lbrace T\in \mathcal {T}_h: u_j(\varvec{x})=a_j\quad \text { or }\quad u_j(\varvec{x})=b_j \quad \forall x\in T \rbrace ,\\ \mathcal {T}_{h,2}^j= & {} \lbrace T\in \mathcal {T}_h: a_j< u_j(\varvec{x}) < b_j \quad \forall x\in T \rbrace ,\quad \mathcal {T}_{h,3}^j \ = \ \mathcal {T}_h \setminus (\mathcal {T}_{h,1}^j \cup \mathcal {T}_{h,2}^j). \end{aligned}$$

Definition (3.36) implies that for any $\tilde{\varvec{u}}_h \in \mathbf {U}_{h,\mathrm {ad}}$, one has (cf. [14, Lemma 2.1]):

3.37 $$\begin{aligned} (\varvec{w}+\lambda \varvec{u}, \tilde{\varvec{u}}-\tilde{\varvec{u}}_h)_{0,\varOmega } \ge 0 \qquad \forall \tilde{\varvec{u}} \in \mathbf {U}_{\mathrm {ad}}. \end{aligned}$$

On the other hand, the following assumption will be instrumental in the subsequent analysis. There exists a positive constant *C* independent of *h* such that

3.38 $$\begin{aligned} \sum \limits _{j=1}^d\sum \limits _{T\in \mathcal {T}_{h,3}^j} |T| \le Ch. \end{aligned}$$

A similar assumption has been employed in [41–43, 48].

We will first focus on error bounds for the control field under piecewise linear discretisation. Before proceeding we state an auxiliary result, whose proof can be found in [41].

#### Lemma 7

Assume (3.38) and that $\varvec{w}\in \mathbf {W}^{1,\infty }(\varOmega )$. Then, there exists $C>0$ independent of *h* such that

$$\begin{aligned} \bigl |(\varvec{w}+\lambda \varvec{u},\tilde{\varvec{u}}_h-\varvec{u})_{0,\varOmega }\bigr | \le \frac{C}{\lambda } h^3 \left\| \nabla \varvec{w}\right\| _{\infty ,\varOmega }^2, \end{aligned}$$

for any $$\tilde{\varvec{u}}_h\in \mathbf {U}_{h,\mathrm {ad}}$$.

The main result in this section is stated as follows.

#### Theorem 2

Let $\varvec{u}\in \mathbf {U}_{\mathrm {ad}}$ be the solution of (1.1)–(1.4) and $\varvec{u}_h \in \mathbf {U}_{h,\mathrm {ad}}$ be the solution of (2.16)–(2.20), under piecewise linear control discretisation. Then, there exists $C>0$ independent of *h* but depending on $\lambda$ such that

$$\begin{aligned} \left\| \varvec{u}-\varvec{u}_h\right\| _{0,\varOmega } \le Ch^{3/2} \left\| \nabla \varvec{w}\right\| _{\infty ,\varOmega }. \end{aligned}$$

#### Proof

Testing the continuous and discrete variational inequalities against $\varvec{u}_h \in \mathbf {U}_{h,\mathrm {ad}}\subset \mathbf {U}_{\mathrm {ad}}$ and $\tilde{\varvec{u}}_h \in \mathbf {U}_{h,\mathrm {ad}}$, respectively, and adding them, leads to

$$\begin{aligned} (\varvec{w}+\lambda \varvec{u},\varvec{u}_h-\varvec{u})_{0,\varOmega }+(\varvec{w}_h+\lambda \varvec{u}_h,\tilde{\varvec{u}}_h-\varvec{u}_h)_{0,\varOmega } \ge 0. \end{aligned}$$

Addition and subtraction of $\tilde{\varvec{u}}_h$ in the first term above yields

$$\begin{aligned} \lambda (\varvec{u}-\varvec{u}_h,\varvec{u}_h-\tilde{\varvec{u}}_h)_{0,\varOmega }+(\varvec{w}-\varvec{w}_h,\varvec{u}_h-\tilde{\varvec{u}}_h)_{0,\varOmega }+(\varvec{w}+\lambda \varvec{u},\tilde{\varvec{u}}_h-\varvec{u})_{0,\varOmega } \ge 0, \end{aligned}$$

and after rearranging terms we obtain

3.39 $$\begin{aligned} \lambda \left\| \varvec{u}-\varvec{u}_h\right\| _{0,\varOmega }^2\le & {} \lambda (\varvec{u}-{\varvec{u}}_h,\varvec{u}-\tilde{\varvec{u}}_h)_{0,\varOmega }+(\varvec{w}-\varvec{w}_h,\varvec{u}_h-\varvec{u})_{0,\varOmega }+(\varvec{w}-\varvec{w}_h,\varvec{u}-\tilde{\varvec{u}}_h)_{0,\varOmega } \nonumber \\&+\,(\varvec{w}+\lambda \varvec{u},\tilde{\varvec{u}}_h-\varvec{u})_{0,\varOmega }. \end{aligned}$$

In view of estimating the term $\left\| \varvec{u}-\tilde{\varvec{u}}_h\right\| _{0,\varOmega }$, we proceed to rewrite it as

3.40 $$\begin{aligned} \begin{aligned} \left\| \varvec{u}-\tilde{\varvec{u}}_h\right\| _{0,\varOmega }^2&= \sum \limits _{j=1}^d \sum \limits _{T\in \mathcal {T}_h}\left\| u_j-\tilde{u}_{j,h}\right\| _{0,T}^2 \\&= \sum \limits _{j=1}^d\sum \limits _{T\in \mathcal {T}_{h,2}^j}\left\| u_j-\tilde{u}_{j,h}\right\| _{0,T}^2 +\sum \limits _{j=1}^d \sum \limits _{T\in \mathcal {T}_{h,3}^j}\left\| u_j-\tilde{u}_{j,h}\right\| _{0,T}^2 \\&=: T_1 + T_2, \end{aligned} \end{aligned}$$

where we have used that $\tilde{u}_{j,h}=u_j$ on $\mathcal {T}_{h,1}^j$, and hence $\sum \limits _{T\in \tau _{h,1}^j}\left\| u_j-\tilde{u}_{j,h}\right\| _{0,T}^2=0$, for $j=1,\ldots ,d$. In order to bound $T_1$ we use the relation $u_j=\frac{-1}{\lambda }w_j$ on all triangles $T\in \mathcal {T}_{h,2}^j$, to obtain

$$\begin{aligned} \sum \limits _{j=1}^d\sum \limits _{T\in \mathcal {T}_{h,2}^j}\left\| u_i-\tilde{I}_h u_i\right\| _{0,T}^2 \le Ch^4\sum \limits _{j=1}^d\sum \limits _{T\in \mathcal {T}_{h,2}^j}\left\| \nabla ^2 u_j\right\| _{0,T}^2 \le \frac{C}{\lambda ^2}h^4 \sum \limits _{j=1}^d\left\| \nabla ^2 w_j\right\| _{0,\varOmega }^2, \end{aligned}$$

whereas for $T_2$, we employ the projection property (1.11) together with (3.38) to get

$$\begin{aligned} \sum \limits _{j=1}^d\sum \limits _{T\in \mathcal {T}_{h,3}^j}\left\| u_j-\tilde{I}_h u_j\right\| _{0,T}^2&\le C\sum \limits _{j=1}^d\sum \limits _{T\in \mathcal {T}_{h,3}^j}|T|\left\| u_j-\tilde{I}_h u_j\right\| _{L^\infty (T)}^2\\&\le Ch^3 \sum \limits _{j=1}^d\left\| \nabla u_j\right\| _{\infty ,\varOmega }^2 \le \frac{C}{\lambda ^2}h^3\sum \limits _{j=1}^d \left\| \nabla w_j\right\| _{\infty ,\varOmega }^2. \end{aligned}$$

Inserting the bounds of $T_1$ and $T_2$ in (3.40) we arrive at

3.41 $$\begin{aligned} \left\| \varvec{u}-\tilde{\varvec{u}}_h\right\| _{0,\varOmega } \le \frac{C}{\lambda }h^2 \sum \limits _{j=1}^d\left\| \nabla ^2 w_j\right\| + \frac{C}{\lambda }h^{3/2}\sum \limits _{j=1}^d \left\| \nabla w_j\right\| _{\infty ,\varOmega }. \end{aligned}$$

Finally, applying Cauchy–Schwarz and Young’s inequalities, the estimates (3.24), (3.41), and Lemmas 5 and 7 into (3.39), we readily obtain the required result. $\square$

We now turn to the $\mathbf {L}^2-$error analysis for the control field under element-wise constant discretisation. The main idea follows from [14], using an $\mathbf {L}^2-$projection $\varPi _0: \mathbf {L}^2(\varOmega ) \longrightarrow \mathbf {U}_{h,0}$ that has the following property: there exists a positive constant *C* independent of *h* such that

3.42 $$\begin{aligned} \left\| \varvec{u}-\varPi _0 \varvec{u}\right\| _{0,\varOmega } \le Ch\left\| \varvec{u}\right\| _{1,\varOmega }. \end{aligned}$$

#### Theorem 3

Let $\varvec{u}$ be the unique solution of (1.1)–(1.4) and $\varvec{u}_h$ be the unique control, solution of the discrete problem (2.16)–(2.20) under an element-wise constant discretisation. Then there exists a positive constant *C* independent of *h* but dependent on $\lambda$ such that

$$\begin{aligned} \left\| \varvec{u}-\varvec{u}_h\right\| _{0,\varOmega } \le Ch\left\| \varvec{u}\right\| _{1,\varOmega }. \end{aligned}$$

#### Proof

Since $\varPi _0 \mathbf {U}_{\mathrm {ad}}\subset \mathbf {U}_{h,\mathrm {ad}}$, the continuous and discrete optimality conditions readily imply that

$$\begin{aligned} (\varvec{w}+\lambda \varvec{u}, \varvec{u}_h - \varvec{u})_{0,\varOmega }+(\varvec{w}_h+\lambda \varvec{u}_h, \varPi _0 \varvec{u}- \varvec{u}_h)_{0,\varOmega } \ge 0. \end{aligned}$$

Adding and subtracting $\varvec{u}$, and rearranging terms, we then obtain

$$\begin{aligned} \lambda \left\| \varvec{u}-\varvec{u}_h\right\| _{0,\varOmega }^2 \le (\varvec{w}-\varvec{w}_h, \varvec{u}_h-\varvec{u})_{0,\varOmega }+(\varvec{w}_h+\lambda \varvec{u}_h, \varPi _0 \varvec{u}-\varvec{u})_{0,\varOmega }, \end{aligned}$$

and since $\varPi _0$ is an orthogonal projection and $\varvec{u}_h \in \mathbf {U}_{h,\mathrm {ad}}$, then the term $\lambda ( \varvec{u}_h, \varPi _0 \varvec{u}-\varvec{u})_{0,\varOmega }$ vanishes to give

3.43 $$\begin{aligned} \lambda \left\| \varvec{u}-\varvec{u}_h\right\| _{0,\varOmega }^2 \le (\varvec{w}-\varvec{w}_h, \varvec{u}_h-\varvec{u})_{0,\varOmega }+(\varvec{w}_h, \varPi _0 \varvec{u}-\varvec{u})_{0,\varOmega }=: I_1+I_2. \end{aligned}$$

For the first term, we use (3.24) to get

$$\begin{aligned} I_1 \le Ch^2\left\| \varvec{u}-\varvec{u}_h\right\| _{0,\varOmega } + Ch\left\| \varvec{u}-\varvec{u}_h\right\| _{0,\varOmega }^2, \end{aligned}$$

whereas a bound for $I_2$ follows from the orthogonality of $\varPi _0$:

$$\begin{aligned} I_2= & {} (\varvec{w}_h- \varPi _0 \varvec{w}_h, \varPi _0 \varvec{u}-\varvec{u})_{0,\varOmega } \le \left\| \varvec{w}_h-\varPi _0 \varvec{w}_h\right\| _{0,\varOmega }\left\| \varPi _0 \varvec{u}-\varvec{u}\right\| _{0,\varOmega }\\\le & {} Ch \left| \left| \left| \varvec{w}_h\right| \right| \right| _{2,h} \left\| \varPi _0 \varvec{u}-\varvec{u}\right\| _{0,\varOmega }. \end{aligned}$$

It is left to show that $\varvec{w}_h$ is uniformly bounded, which can be readily derived using the coercivity of $A_h(\cdot ,\cdot )$ and $c_h(\cdot ,\cdot )$ and the uniform boundedness of $\mathbf {U}_{h,\mathrm {ad}}$:

$$\begin{aligned} \left| \left| \left| \varvec{w}_h\right| \right| \right| _{2,h} \le C\left( \left\| \varvec{u}_h\right\| _{0,\varOmega }+\left\| \varvec{f}\right\| _{0,\varOmega }+\left\| \varvec{y}_d\right\| _{0,\varOmega } \right) \le C. \end{aligned}$$

Substituting the bounds for $I_1$ and $I_2$ in (3.43), and using (3.42) the desired result follows. $\square$

### $\mathbf {L}^2$-Error Estimates for Velocity Under Full Discretisation of Control

The main result in this section is given as follows (see similar ideas, based on duality arguments also applied in [43, 50]).

#### Theorem 4

Let $( \varvec{y}, \varvec{w})$ be the state and co-state velocities, solutions of (1.1)–(1.4), and let $(\varvec{y}_h, \varvec{w}_h)$ be their DFV approximations under piecewise linear (or piecewise constant) discretisation of control. Then

$$\begin{aligned} \left\| \varvec{y}-\varvec{y}_h\right\| _{0,\varOmega }&\le Ch^2 [ \left\| \varvec{y}\right\| _{2,\varOmega }+\left\| p\right\| _{1,\varOmega } +\left\| \varvec{u}\right\| _{1,\varOmega } +\left\| \varvec{f}\right\| _{1,\varOmega } ],\\ \left\| \varvec{w}-\varvec{w}_h\right\| _{0,\varOmega }&\le Ch^2 [ \left\| \varvec{y}\right\| _{2,\varOmega }+\left\| p\right\| _{1,\varOmega } +\left\| \varvec{u}\right\| _{1,\varOmega } +\left\| \varvec{f}\right\| _{1,\varOmega }+\left\| \varvec{w}\right\| _{2,\varOmega }+\left\| r\right\| _{1,\varOmega } \\&\qquad + \left\| \varvec{y}\right\| _{1,\varOmega } +\left\| \varvec{y}_d\right\| _{1,\varOmega } ]. \end{aligned}$$

#### Proof

We start by splitting the total error and applying triangle inequality as:

3.44 $$\begin{aligned} \left\| \varvec{y}-\varvec{y}_h\right\| _{0,\varOmega } \le \left\| \varvec{y}-\varvec{y}_h(\varvec{u})\right\| _{0,\varOmega }+\left\| \varvec{y}_h(\varvec{u})-\varvec{y}_h(\varPi _h \varvec{u})\right\| _{0,\varOmega }+\left\| \varvec{y}_h(\varPi _h \varvec{u})-\varvec{y}_h\right\| _{0,\varOmega }, \end{aligned}$$

where $\varPi _h$ represents the $\mathbf {L}^2-$projection operator onto the discrete control space $\mathbf {U}_h$. Next, let $(\tilde{\varvec{w}}_h,\tilde{r}_h) \in \mathbf {V}_h\times Q_h$ be the unique solution of the auxiliary discrete dual Brinkman problem

3.45 $$\begin{aligned} A_h(\tilde{\varvec{w}}_h,\tilde{\varvec{z}}_h)+c_h(\tilde{\varvec{w}}_h,\tilde{\varvec{z}}_h)-B_h(\tilde{\varvec{z}}_h,\tilde{r}_h)&=(\gamma \tilde{\varvec{z}}_h,\varvec{y}_h(\varvec{u})-\varvec{y}_h(\varPi _h \varvec{u}))_{0,\varOmega }\quad \forall \tilde{\varvec{z}}_h\in \mathbf {V}_h, \end{aligned}$$

3.46 $$\begin{aligned} B_h(\tilde{\varvec{w}}_h,\tilde{\psi }_h)&=0 \quad \forall \tilde{\psi }_h \in Q_h. \end{aligned}$$

We then choose $\tilde{\varvec{z}}_h=\tilde{\varvec{w}}_h$ and $\tilde{\psi }_h=\tilde{r}_h$ in (3.45) and (3.46), respectively, next we add the result, and we use the coercivity properties (2.8) and (2.12), to derive that

3.47 $$\begin{aligned} \left| \left| \left| \tilde{\varvec{w}}_h\right| \right| \right| _{2,h}\le C \left\| \varvec{y}_h(\varvec{u})-\varvec{y}_h(\varPi _h \varvec{u})\right\| _{0,\varOmega }. \end{aligned}$$

After testing (3.45)–(3.46) against $\tilde{\varvec{z}}_h=\varvec{y}_h(\varvec{u})-\varvec{y}_h(\varPi _h \varvec{u})$ and $\tilde{\psi }_h=p_h(\varvec{u})-p_h(\varPi _h \varvec{u})$, respectively, and adding the result, we obtain

3.48 $$\begin{aligned}&A_h(\tilde{\varvec{w}}_h,\varvec{y}_h(\varvec{u})-\varvec{y}_h(\varPi _h \varvec{u}))+c_h(\tilde{\varvec{w}}_h,\varvec{y}_h(\varvec{u})-\varvec{y}_h(\varPi _h \varvec{u}))-B_h(\varvec{y}_h(\varvec{u})-\varvec{y}_h(\varPi _h \varvec{u}),\tilde{r}_h)\nonumber \\&\quad -B_h(\tilde{\varvec{w}}_h,p_h(\varvec{u})-p_h(\varPi _h \varvec{u})) =(\gamma (\varvec{y}_h(\varvec{u})-\varvec{y}_h(\varPi _h \varvec{u})),\varvec{y}_h(\varvec{u})-\varvec{y}_h(\varPi _h \varvec{u}))_{0,\varOmega }.\nonumber \\ \end{aligned}$$

In addition, employing the discrete state equation for $\varvec{y}_h(\varvec{u})$ and $\varvec{y}_h(\varPi _h \varvec{u})$, we obtain

3.49 $$\begin{aligned}&A_h(\varvec{y}_h(\varvec{u})-\varvec{y}_h(\varPi _h \varvec{u}),\tilde{\varvec{w}}_h)+c_h(\varvec{y}_h(\varvec{u})-\varvec{y}_h(\varPi _h \varvec{u}),\tilde{\varvec{w}}_h)-B_h(\tilde{\varvec{w}}_h,p_h(\varvec{u})-p_h(\varPi _h \varvec{u}))\nonumber \\&\quad -B_h(\varvec{y}_h(\varvec{u})-\varvec{y}_h(\varPi _h \varvec{u}),\tilde{r}_h)=(\varvec{u}-\varPi _h \varvec{u},\gamma \tilde{\varvec{w}}_h)_{0,\varOmega }. \end{aligned}$$

We then proceed to subtract (3.49) from (3.48) and to rearrange terms, to arrive at

$$\begin{aligned}&(\gamma (\varvec{y}_h(\varvec{u})-\varvec{y}_h(\varPi _h \varvec{u})),\varvec{y}_h(\varvec{u})-\varvec{y}_h(\varPi _h \varvec{u}))_{0,\varOmega } \\&\quad = A_h(\tilde{\varvec{w}}_h,\varvec{y}_h(\varvec{u})-\varvec{y}_h(\varPi _h \varvec{u}))-A_h(\varvec{y}_h(\varvec{u})-\varvec{y}_h(\varPi _h \varvec{u}),\tilde{\varvec{w}}_h)\\&\qquad +c_h(\tilde{\varvec{w}}_h,\varvec{y}_h(\varvec{u})-\varvec{y}_h(\varPi _h \varvec{u}))\\&\qquad -c_h(\varvec{y}_h(\varvec{u})-\varvec{y}_h(\varPi _h \varvec{u}),\tilde{\varvec{w}}_h) +(\varvec{u}-\varPi _h \varvec{u},\gamma \tilde{\varvec{w}}_h)_{0,\varOmega }. \end{aligned}$$

Using the definition of the norm $\left| \left| \left| \cdot \right| \right| \right| _{0,h}$ and its equivalence with the norm $\Vert \cdot \Vert _{0,\varOmega }$ we find that

$$\begin{aligned}&\left\| \varvec{y}_h(\varvec{u})-\varvec{y}_h(\varPi _h \varvec{u})\right\| _{0,\varOmega }^2 \\&\quad \le (\varvec{u}-\varPi _h \varvec{u},\gamma \tilde{\varvec{w}}_h)_{0,\varOmega }+|A_h(\tilde{\varvec{w}}_h,\varvec{y}_h(\varvec{u})-\varvec{y}_h(\varPi _h \varvec{u}))-A_h(\varvec{y}_h(\varvec{u})-\varvec{y}_h(\varPi _h \varvec{u}),\tilde{\varvec{w}}_h)|\\&\qquad +|c_h(\tilde{\varvec{w}}_h,\varvec{y}_h(\varvec{u})-\varvec{y}_h(\varPi _h \varvec{u}))-c_h(\varvec{y}_h(\varvec{u})-\varvec{y}_h(\varPi _h \varvec{u}),\tilde{\varvec{w}}_h)|. \end{aligned}$$

By virtue of the properties of $\varPi _h$ applied in the above inequality, we can assert that

3.50 $$\begin{aligned} \left\| \varvec{y}_h(\varvec{u})-\varvec{y}_h(\varPi _h \varvec{u})\right\| _{0,\varOmega }^2&\le (\varvec{u}-\varPi _h \varvec{u},\gamma \tilde{\varvec{w}}_h-\tilde{\varvec{w}}_h)_{0,\varOmega }+ (\varvec{u}-\varPi _h \varvec{u}, \tilde{\varvec{w}}_h-\varPi _h \tilde{\varvec{w}}_h)_{0,\varOmega }\nonumber \\&\qquad +|A_h(\tilde{\varvec{w}}_h,\varvec{y}_h(\varvec{u})-\varvec{y}_h(\varPi _h \varvec{u}))-A_h(\varvec{y}_h(\varvec{u})-\varvec{y}_h(\varPi _h \varvec{u}),\tilde{\varvec{w}}_h)|\nonumber \\&\qquad +|c_h(\tilde{\varvec{w}}_h,\varvec{y}_h(\varvec{u})-\varvec{y}_h(\varPi _h \varvec{u}))-c_h(\varvec{y}_h(\varvec{u})-\varvec{y}_h(\varPi _h \varvec{u}),\tilde{\varvec{w}}_h)|\nonumber \\&=: S_1+S_2+S_3+S_4. \end{aligned}$$

Approximation properties of $\gamma$ and the $\mathbf {L}^2-$projection readily yield appropriate bounds for $S_1$ and $S_2$, respectively:

$$\begin{aligned} S_1&\le Ch\left\| \varvec{u}-\varPi _h \varvec{u}\right\| _{0,\varOmega }\left| \left| \left| \tilde{\varvec{w}}_h\right| \right| \right| _{2,h},\quad \text {and} \quad S_2 \le Ch\left\| \varvec{u}-\varPi _h \varvec{u}\right\| _{0,\varOmega }\left| \left| \left| \tilde{\varvec{w}}_h\right| \right| \right| _{2,h}. \end{aligned}$$

Then, a direct application of (3.47) yields

$$\begin{aligned} S_1+S_2 \le Ch\left\| \varvec{u}-\varPi _h \varvec{u}\right\| _{0,\varOmega }\left\| \varvec{y}_h(\varvec{u})-\varvec{y}_h(\varPi _h \varvec{u})\right\| _{0,\varOmega }. \end{aligned}$$

We next use relations (2.11), (2.13) and (3.47) to obtain

$$\begin{aligned} S_3+S_4\le & {} Ch\left| \left| \left| \varvec{y}_h(\varvec{u})-\varvec{y}_h(\varPi _h \varvec{u})\right| \right| \right| _{2,h}\left| \left| \left| \tilde{\varvec{w}}_h\right| \right| \right| _{2,h}\\\le & {} Ch\left\| \varvec{u}-\varPi _h \varvec{u}\right\| _{0,\varOmega }\left\| \varvec{y}_h(\varvec{u})-\varvec{y}_h(\varPi _h \varvec{u})\right\| _{0,\varOmega }. \end{aligned}$$

Consequently, substituting the estimates for the terms $S_1$, $S_2$, $S_3$ and $S_4$ back into (3.50), one straightforwardly arrives at

3.51 $$\begin{aligned} \left\| \varvec{y}_h(\varvec{u})-\varvec{y}_h(\varPi _h \varvec{u})\right\| _{0,\varOmega }\le Ch\left\| \varvec{u}-\varPi _h \varvec{u}\right\| _{0,\varOmega }. \end{aligned}$$

The third term in (3.44) is bounded using (2.7) and proceeding as in the proof of Lemma 4:

3.52 $$\begin{aligned} \left\| \varvec{y}_h(\varPi _h \varvec{u})-\varvec{y}_h\right\| _{0,\varOmega } \le \left| \left| \left| \varvec{y}_h(\varPi _h \varvec{u})-\varvec{y}_h\right| \right| \right| _{2,h} \le C\left\| \varPi _h \varvec{u}-\varvec{u}_h\right\| _{0,\varOmega }. \end{aligned}$$

Using the discrete variational inequality along with the projection property of $\varPi _h$ and (3.37), we have the following relation

3.53 $$\begin{aligned} \lambda \left\| \varPi _h \varvec{u}-\varvec{u}_h\right\| _{0,\varOmega }^2= & {} \lambda (\varvec{u}-\varvec{u}_h,\varPi _h \varvec{u}-\varvec{u}_h)_{0,\varOmega } \le (\varvec{w}-\varvec{w}_h,\varvec{u}_h-\varPi _h \varvec{u})_{0,\varOmega }\nonumber \\= & {} (\varvec{w}-\varvec{w}_h({\varvec{u}}),\varvec{u}_h-\varPi _h \varvec{u})_{0,\varOmega }+(\varvec{w}_h({\varvec{u}})\nonumber \\&-\,\varvec{w}_h({\varvec{y}}_h(\varPi _h {\varvec{u}})),\varvec{u}_h-\varPi _h \varvec{u})_{0,\varOmega }\nonumber \\&+\,(\varvec{w}_h({\varvec{y}}_h(\varPi _h {\varvec{u}}))-\varvec{w}_h,\varvec{u}_h-\varPi _h \varvec{u})_{0,\varOmega }\nonumber \\= & {} (\varvec{w}-\varvec{w}_h({\varvec{u}}),\varvec{u}_h-\varPi _h \varvec{u})_{0,\varOmega }+(\varvec{w}_h({\varvec{u}})\nonumber \\&-\,\varvec{w}_h({\varvec{y}}_h(\varPi _h {\varvec{u}})),\varvec{u}_h-\varPi _h \varvec{u})_{0,\varOmega }\nonumber \\&+\,(\varvec{w}_h({\varvec{y}}_h(\varPi _h {\varvec{u}}))-\varvec{w}_h-\gamma (\varvec{w}_h({\varvec{y}}_h(\varPi _h {\varvec{u}}))-\varvec{w}_h),\varvec{u}_h-\varPi _h \varvec{u})_{0,\varOmega }\nonumber \\&+\,(\gamma (\varvec{w}_h({\varvec{y}}_h(\varPi _h {\varvec{u}}))-\varvec{w}_h),\varvec{u}_h-\varPi _h \varvec{u})_{0,\varOmega }\nonumber \\= & {} J_1+J_2+J_3+J_4. \end{aligned}$$

Next, we use Cauchy–Schwarz inequality and (3.23) to bound the first term:

$$\begin{aligned} J_1 \le \left\| \varvec{w}-\varvec{w}_h(u)\right\| _{0,\varOmega }\left\| \varvec{u}_h-\varPi _h \varvec{u}\right\| _{0,\varOmega }\le Ch^2\left\| \varvec{u}_h-\varPi _h \varvec{u}\right\| _{0,\varOmega }. \end{aligned}$$

For $J_2$, an application of Lemma 4 and (3.51) suffices to get

$$\begin{aligned} J_2 \le \left\| \varvec{y}_h(\varvec{u})-\varvec{y}_h(\varPi _h \varvec{u})\right\| _{0,\varOmega }\left\| \varvec{u}_h-\varPi _h \varvec{u}\right\| _{0,\varOmega }\le Ch\left\| \varvec{u}-\varPi _h \varvec{u}\right\| _{0,\varOmega }\left\| \varvec{u}_h-\varPi _h \varvec{u}\right\| _{0,\varOmega }. \end{aligned}$$

To bound the third term we use the approximation property of $\gamma$ and Lemma 4

$$\begin{aligned} J_3\le & {} Ch\left| \left| \left| \varvec{w}_h(\varvec{y}_h(\varPi _h \varvec{u}))-\varvec{w}_h\right| \right| \right| _{2,h}\left\| \varvec{u}_h-\varPi _h \varvec{u}\right\| _{0,\varOmega }\\\le & {} Ch\left\| \varvec{y}_h(\varPi _h \varvec{u})-\varvec{y}_h\right\| _{0,\varOmega }\left\| \varvec{u}_h-\varPi _h \varvec{u}\right\| _{0,\varOmega } \le Ch\left\| \varvec{u}_h-\varPi _h \varvec{u}\right\| _{0,\varOmega }^2. \end{aligned}$$

Proceeding analogously to the proof of Lemma 6, using (2.11) and (2.13), the last term of the expression (3.53) can be estimated as

$$\begin{aligned} J_4\le & {} A_h(\varvec{y}_h-\varvec{y}_h(\varPi _h \varvec{u}), \varvec{w}_h(\varPi _h \varvec{u})-{\varvec{w}}_h)-A_h(\varvec{w}_h(\varPi _h \varvec{u})-{\varvec{w}}_h,\varvec{y}_h-\varvec{y}_h(\varPi _h \varvec{u}))\\&+\,c_h(\varvec{y}_h-\varvec{y}_h(\varPi _h \varvec{u}), \varvec{w}_h(\varPi _h \varvec{u})-{\varvec{w}}_h)-c_h(\varvec{w}_h(\varPi _h \varvec{u})-{\varvec{w}}_h,\varvec{y}_h-\varvec{y}_h(\varPi _h \varvec{u}))\\\le & {} Ch\left| \left| \left| \varvec{y}_h-\varvec{y}_h(\varPi _h \varvec{u})\right| \right| \right| _{2,h}\left| \left| \left| \varvec{w}_h(\varPi _h \varvec{u})-{\varvec{w}}_h\right| \right| \right| _{2,h} \le Ch \left\| \varvec{u}_h-\varPi _h \varvec{u}\right\| _{0,\varOmega }^2. \end{aligned}$$

Plugging the bounds for $J_1, J_2, J_3$ and $J_4$ in (3.53), putting (3.51) and (3.52) into (3.44), and using interpolation estimate $\left\| \varvec{u}-\varPi _h \varvec{u}\right\| _{0,\varOmega } \le Ch\left\| \varvec{u}\right\| _{1,\varOmega }$ along with Lemma 5; we can assert that

3.54 $$\begin{aligned} \left\| \varvec{y}-\varvec{y}_h\right\| _{0,\varOmega }\le Ch^2 \left[ \left\| \varvec{y}\right\| _{2,\varOmega }+\left\| p\right\| _{1,\varOmega } +\left\| \varvec{u}\right\| _{1,\varOmega } +\left\| \varvec{f}\right\| _{1,\varOmega } \right] . \end{aligned}$$

Finally, splitting the co-state velocity error as $\varvec{w}-\varvec{w}_h=\varvec{w}-\varvec{w}_h(\varvec{y})+\varvec{w}_h(y)-\varvec{w}_h$, using triangle inequality and Lemmas 4,5, and relation (3.54), we get the second desired estimate

$$\begin{aligned} \left\| \varvec{w}-\varvec{w}_h\right\| _{0,\varOmega }\le \left\| \varvec{w}-\varvec{w}_h(\varvec{y})\right\| _{0,\varOmega }+\left\| \varvec{w}_h(\varvec{y})-\varvec{w}_h\right\| _{0,\varOmega } \le \left\| \varvec{w}-\varvec{w}_h(\varvec{y})\right\| _{0,\varOmega }+\left\| \varvec{y}-\varvec{y}_h\right\| _{0,\varOmega }. \end{aligned}$$

$$\square $$

### Error Bounds in the Energy Norm

#### Theorem 5

Let $( \varvec{y}, \varvec{w}, p, r )$ be the state and co-state velocities, and pressures, solutions of (1.1)–(1.4), and let $(\varvec{y}_h, \varvec{w}_h, p_h, r_h)$ be their DFV approximations. Then

$$\begin{aligned} \left| \left| \left| \varvec{y}-\varvec{y}_h\right| \right| \right| _{2,h}+\left\| p-p_h\right\| _{0,\varOmega } \le Ch\left( \left\| \varvec{y}\right\| _{2,\varOmega }+\left\| p\right\| _{1,\varOmega }\right) \\ \left| \left| \left| \varvec{w}-\varvec{w}_h\right| \right| \right| _{2,h}+\left\| r-r_h\right\| _{0,\varOmega } \le Ch\left( \left\| \varvec{w}\right\| _{2,\varOmega }+\left\| r\right\| _{1,\varOmega }\right) . \end{aligned}$$

#### Proof

Using (3.5) and (3.6), applying triangle inequality and Lemma 4, we obtain

$$\begin{aligned}&\left| \left| \left| \varvec{y}-\varvec{y}_h\right| \right| \right| _{2,h}+\left\| p-p_h\right\| _{0,\varOmega } \\&\qquad \le \left| \left| \left| \varvec{y}-\varvec{y}_h(\varvec{u})\right| \right| \right| _{2,h}+\left| \left| \left| \varvec{y}_h(\varvec{u})-\varvec{y}_h\right| \right| \right| _{2,h}+\left\| p-p_h(\varvec{u})\right\| _{0,\varOmega }+\left\| p_h(\varvec{u})-p_h\right\| _{0,\varOmega }\\&\qquad \le \left| \left| \left| \varvec{y}-\varvec{y}_h(\varvec{u})\right| \right| \right| _{2,h}+\left\| p-p_h(\varvec{u})\right\| _{0,\varOmega }+C\left\| \varvec{u}-\varvec{u}_h\right\| _{0,\varOmega }, \end{aligned}$$

and

$$\begin{aligned}&\left| \left| \left| \varvec{w}-\varvec{w}_h\right| \right| \right| _{2,h}+\left\| r-r_h\right\| _{0,\varOmega } \\&\qquad \le \left| \left| \left| \varvec{w}-\varvec{w}_h(\varvec{y})\right| \right| \right| _{2,h}+\left| \left| \left| \varvec{w}_h(\varvec{y})-\varvec{w}_h\right| \right| \right| _{2,h}+\left\| r-r_h(\varvec{y})\right\| _{0,\varOmega }+\left\| r_h(\varvec{y})-r_h\right\| _{0,\varOmega } \\&\qquad \le \left| \left| \left| \varvec{w}-\varvec{w}_h(\varvec{y})\right| \right| \right| _{2,h}+\left\| r-r_h(\varvec{y})\right\| _{0,\varOmega }+C\left\| \varvec{y}-\varvec{y}_h\right\| _{0,\varOmega }. \end{aligned}$$

The proof follows after combining Lemma 5 with the bounds for $\left\| \varvec{u}-\varvec{u}_h\right\| _{0,\varOmega }$ and $\left\| \varvec{y}-\varvec{y}_h\right\| _{0,\varOmega }$. $\square$

## Numerical Experiments

In this section we present a set of numerical examples to illustrate the theoretical results previously described. For sake of completeness, before jumping into the tests we provide some details about the implementation and algorithms for the efficient numerical realisation of the DFV method applied to the optimal control of Brinkman equations.

**Implementation Aspects** We will use the well-known active set strategy (proposed in [6]) involving primal and dual variables (see also [25, 44] for its application in Stokes flow). The principle is to approximate the constrained optimal control problem by a sequence of unconstrained problems, using active sets as summarised in Algorithm 1. By $\varvec{u}^n_{h},\varvec{w}^n_h$ we will denote the optimal control and adjoint velocity, solutions to the discrete problem (2.16)–(2.20) at the current iteration. Also, the control constraints are $\varvec{a}=(a_1,..a_d)^T$ and $\varvec{b}=(b_1,..b_d)^T$.

Let $\lbrace \mathbf {\varvec{\phi }_i}^{\,} \rbrace _{i=1}^N$, $\lbrace \xi _i \rbrace _{i=1}^L$, $\lbrace \mathbf {\varvec{\psi }_i}^{\,} \rbrace _{i=1}^M$ be the basis functions for $\mathbf {V}_h$, $Q_h$, and $\mathbf {U}_h$, respectively, whereas the space $\mathbf {V}_h^*$ is spanned by $\lbrace \mathbf {\varvec{\phi }^*_i}^{\,} \rbrace _{i=1}^N$, with (made precise here for $d=3$)

$$\begin{aligned} \mathbf {\varvec{\phi }^*_i}^{\,}(\varvec{x})= \{\chi _{T^*_i}(1,0,0),\chi _{T^*_i}(0,1,0),\chi _{T^*_i}(0,0,1)\}, \end{aligned}$$

where $\chi _{T^*_i}$ is the characteristic function assuming the value 1 on $T_i^*\in \mathcal {T}_h^*$ and zero elsewhere.
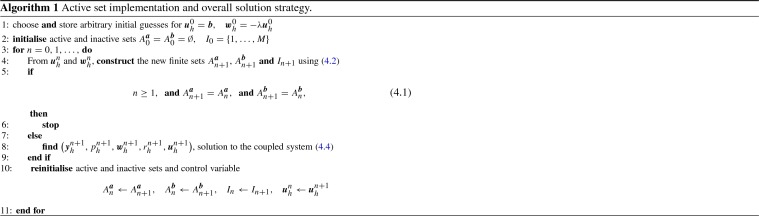


We next proceed to define the discrete active and inactive sets, based on the degrees of freedom of $\mathbf {U}_h$, as follows

4.2 $$\begin{aligned} \begin{aligned} A_{n+1}^{\varvec{a}}&=\lbrace k\in \lbrace 1,\ldots ,M \rbrace : -w^{n,k}_{j,h}/\lambda < a_j, \text { for any } j\in \{1,\ldots ,d\} \rbrace ,\\ A_{n+1}^{\varvec{b}}&=\lbrace k\in \lbrace 1,\ldots ,M \rbrace : -w^{n,k}_{j,h}/\lambda > b_j, \text { for any } j\in \{1,\ldots ,d\} \rbrace ,\\ I_{n+1}&=\lbrace 1,\ldots ,M \rbrace \setminus (A_{n+1}^{\varvec{a}} \cup A_{n+1}^{\varvec{b}}), \end{aligned} \end{aligned}$$

where, in general, $s^{n,k}_{j,h}$ stands for the discrete value associated with the degree of freedom at position *k*, related to the spatial component *j* of the vector field $\varvec{s}$, at the step *n* of Algorithm 1. By the definition of the optimal control problem, we have that

$$\begin{aligned} \varvec{u}^n_{h}={\left\{ \begin{array}{ll} \varvec{a}^{\,} &{} \text {in}\quad A_{n+1}^{\varvec{a}},\\ -\lambda ^{-1}\varvec{w}^n_{h} &{} \text {in}\quad I_{n+1},\\ \varvec{b}^{\,} &{} \text {in} \quad A_{n+1}^{\varvec{b}}, \end{array}\right. } \end{aligned}$$

and if we further introduce the following characteristic sets

$$\begin{aligned} \chi _{A_{n+1}^{\varvec{a}}(k,k)}={\left\{ \begin{array}{ll} 1 &{} \text {if}\quad k\in A_{n+1}^{\varvec{a}},\\ 0 &{} \text {else}, \end{array}\right. } \quad \chi _{A_{n+1}^{\varvec{b}}(k,k)}={\left\{ \begin{array}{ll} 1 &{} \text {if}\quad k\in A_{n+1}^{\varvec{b}},\\ 0 &{} \text {else}, \end{array}\right. } \end{aligned}$$

then we get

4.3 $$\begin{aligned} \lambda ^{-1} \varvec{w}^n_{h}\left( 1-\chi _{A_{n+1}^{\varvec{a}}}-\chi _{A_{n+1}^{\varvec{b}}}\right) +\varvec{u}^n_{h}=\varvec{a}^{\,} \chi _{A_{n+1}^{\varvec{a}}}+\varvec{b}^{\,}\chi _{A_{n+1}^{\varvec{b}}}. \end{aligned}$$

Finally, we define the matrix blocks

$$\begin{aligned} \mathbb {A}=&[A_h(\mathbf {\varvec{\phi }_i},\mathbf {\varvec{\phi }_j})]_{ i,j \le N},\, \mathbb {C}=[c_h(\mathbf {\varvec{\phi }_i},\mathbf {\varvec{\phi }_j})]_{ i,j \le N},\, \mathbb {B}=[B_h(\xi _i,\mathbf {\varvec{\phi }_j})]_{ i\le L; j\le N},\,\\ \mathbb {M}=&[(\mathbf {\varvec{\phi }_i}, \mathbf {\varvec{\phi }_j^*})_{0,\varOmega }]_{ i,j \le N},\quad \mathbb {G}= [( \mathbf {\varvec{\phi }_i^*}^{\,},\mathbf {\varvec{\psi }_j}^{\,})_{0,\varOmega }]_{ i\le N;1 j\le M},\nonumber \\ \mathbb {D}=&[(\mathbf {\varvec{\psi }_i}^{\,},\mathbf {\varvec{\psi }_j}^{\,})_{0,\varOmega }]_{ i,j \le M},\quad \hat{\mathbb {E}}=\lambda ^{-1}(\mathbf {I}-\chi _{A_{n+1}^{\varvec{a}}}-\chi _{A_{n+1}^{\varvec{b}}}), \end{aligned}$$

along with the vectors

$$\begin{aligned} \mathrm {F}=[(\varvec{f},\mathbf {\varvec{\phi }_i^*}^{\,} )_{0,\varOmega }]_{ i \le N},\quad \mathrm {Y}_d=[(\varvec{y}_d,\mathbf {\varvec{\phi }_i^*}^{\,})_{0,\varOmega }]_{ i \le N},\quad \hat{\mathrm {S}}=[(\varvec{a}^{\,}\chi _{A_{n+1}^{\varvec{a}}}+\varvec{b}^{\,}\chi _{A_{n+1}^{\varvec{b}}},\mathbf {\varvec{\psi }_i}^{\,})_{0,\varOmega }]_{ i \le M}, \end{aligned}$$

so that after testing (4.3) against $\lbrace \mathbf {\varvec{\psi }_i} \rbrace _{i=1}^M$ we end up with the following matrix form of the discrete optimal control problem (2.16)–(2.20):

4.4 $$\begin{aligned} \begin{pmatrix} \mathbb {A}+\mathbb {C}&{} -\mathbb {B}^T&{} \varvec{0}&{} \varvec{0}&{} -\mathbb {G}\\ \mathbb {B}&{} \varvec{0}&{} \varvec{0}&{} \varvec{0}&{} \varvec{0}\\ -\mathbb {M}&{} \varvec{0}&{} \mathbb {A}+\mathbb {C}&{} \mathbb {B}^T&{} \varvec{0}\\ \varvec{0}&{} \varvec{0}&{} -\mathbb {B}&{} \varvec{0}&{} \varvec{0}\\ \varvec{0}&{} \varvec{0}&{} \hat{\mathbb {E}}\mathbb {G}^T&{} \varvec{0}&{} \mathbb {D}\end{pmatrix} \begin{pmatrix} {\mathrm {Y}} \\ {\mathrm {P}} \\ {\mathrm {W}}\\ {\mathrm {R}}\\ {\mathrm {U}} \end{pmatrix}= \begin{pmatrix} {\mathrm {F}} \\ \varvec{0}\\ -{\mathrm {Y}}_d\\ \varvec{0}\\ \hat{\mathrm {S}} \end{pmatrix}, \end{aligned}$$

where $\mathrm {Y}, \mathrm {P}, \mathrm {W}, \mathrm {R}$ and $\mathrm {U}$ are the coefficients in the expansion of $\varvec{y}^{n+1}_{h}$, $p^{n+1}_{h}$, $\varvec{w}^{n+1}_{h}$, $r^{n+1}_{h}$ and $\varvec{u}^{n+1}_{h}$, respectively, and the hats indicate quantities associated with the previous iteration.

**Table 1:** Example 1: convergence history and optimisation iteration count for the approximations of the optimal control of the Brinkman problem

*h*	$e_0(\varvec{y})$	$\mathtt{rate}$	$e_1(\varvec{y})$	$\mathtt{rate}$	$e_0(p)$	$\mathtt{rate}$	$e_0(\varvec{w})$	$\mathtt{rate}$	$e_1(\varvec{w})$	$\mathtt{rate}$	$e_0(r)$	$\mathtt{rate}$	$e_0(\varvec{u})$	$\mathtt{rate}$	it
*Piecewise constant control*															
0.71	0.3025	–	2.2608	–	0.3301	–	0.3025	–	2.2608	–	0.3301	–	0.1824	–	2
0.47	0.1744	1.35	1.5583	0.91	0.3062	0.38	0.1770	1.32	1.5621	0.911	0.3062	0.39	0.1204	1.02	3
0.28	0.0872	1.35	1.0574	0.75	0.1995	0.83	0.0893	1.33	1.0600	0.859	0.1995	0.83	0.0743	0.94	3
0.15	0.0316	1.72	0.6188	0.91	0.1146	0.94	0.0326	1.71	0.6196	0.913	0.1146	0.94	0.0416	0.98	3
0.08	0.0084	2.09	0.3327	0.97	0.0613	0.98	0.0086	2.09	0.3328	0.977	0.0613	0.98	0.0216	1.02	3
0.04	0.0015	2.15	0.1725	0.99	0.0317	0.99	0.0016	2.57	0.1725	0.991	0.0317	0.99	0.0111	1.00	3
0.02	0.0004	2.13	0.0885	0.98	0.0161	0.98	0.0004	2.02	0.0886	0.982	0.0161	0.99	0.0053	1.04	3
0.01	0.0001	2.00	0.0464	0.94	0.0081	0.99	0.0001	2.03	0.0465	0.989	0.0081	0.99	0.0027	1.02	3
*Piecewise linear control*															
0.71	0.3025	–	2.2608	–	0.3301	–	0.3025	–	2.2608	–	0.3301	–	0.1825	–	2
0.47	0.1751	1.34	1.5593	0.91	0.3062	0.38	0.1770	1.32	1.5622	0.91	0.3062	0.38	0.0886	1.78	3
0.28	0.0876	1.35	1.0578	0.75	0.1995	0.83	0.0893	1.33	1.0600	0.75	0.1995	0.83	0.0540	0.96	3
0.15	0.0318	1.72	0.6190	0.91	0.1146	0.94	0.0326	1.71	0.6196	0.91	0.1146	0.94	0.0243	1.36	3
0.08	0.0084	2.08	0.3322	0.97	0.0613	0.98	0.0086	2.09	0.3328	0.97	0.0613	0.98	0.0090	1.55	3
0.04	0.0015	2.17	0.1729	0.99	0.0317	0.99	0.0016	2.17	0.1725	0.99	0.0317	0.99	0.0032	1.56	3
0.02	0.0004	2.13	0.0882	0.98	0.0159	0.99	0.0005	1.99	0.0887	0.99	0.0160	0.99	0.0011	1.55	2
0.01	0.0001	2.01	0.0460	0.94	0.0075	0.94	0.0001	2.02	0.0453	0.97	0.0079	0.99	0.0002	1.57	3

**Fig. 2:**
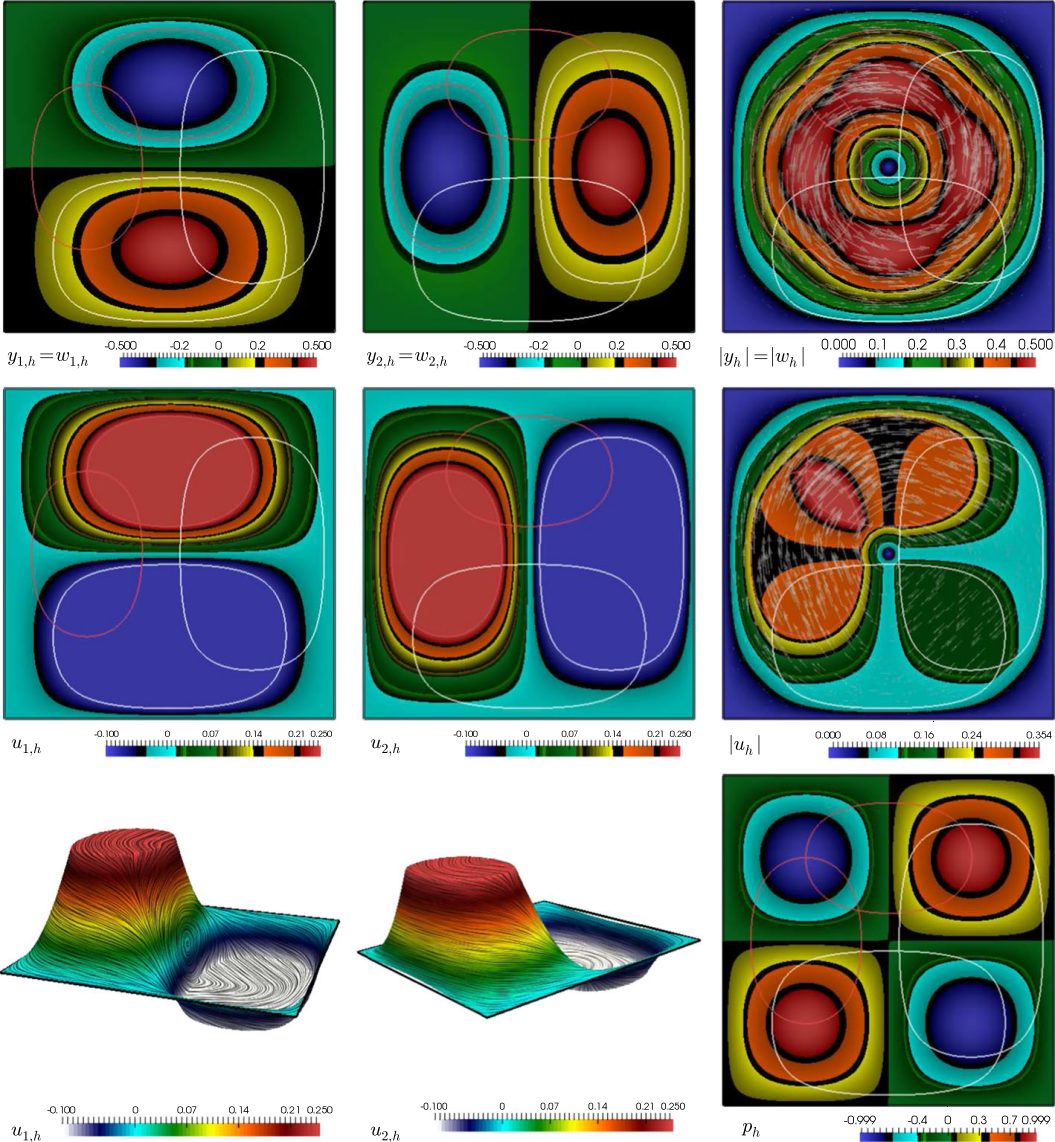
Example 1: DFV approximation of state velocity components and magnitude (top panels), components and magnitude of the control variable; approximated with piecewise linear elements (centre row), and state pressure field (bottom row). Contours of the active sets for $a_1=a_2$ (in white curves) and $b_1=b_2$ (red curves) are displayed (Color figure online)

### Example 1

We start by assessing the experimental convergence of the proposed scheme applied to the optimal control problem (1.1)–(1.4) defined on the unit square $\varOmega =(0,1)^2$. Viscosity, permeability and the weight for the control cost assume the following constant values $\nu =1$, $\mathbf {K}=\mathrm {diag}(1,1.5)$, $\lambda =1$, respectively. The set of admissible controls is characterised by the constants $a_1=a_2=-\frac{1}{10},b_1=b_2=\frac{1}{4}$, and manufactured solutions are explicitly given by

$$\begin{aligned} \varvec{y}= & {} \varvec{w}=\begin{pmatrix} \sin ^2(\pi x_1)\sin (\pi x_2)\cos (\pi x_2)\\ -\sin ^2(\pi x_2)\sin (\pi x_1)\cos (\pi x_1)\end{pmatrix},\ p=-r=\sin (2\pi x_1)\sin (2\pi x_2),\\ \varvec{u}= & {} P_{[\varvec{a},\varvec{b}]}\biggl (\frac{-1}{\lambda } \varvec{w}\biggr ), \end{aligned}$$

(see e.g. [48]) which satisfy the homogeneous Dirichlet boundary conditions under which the analysis was performed. Source term and desired velocity field of the problem are constructed according to these exact solutions, that is, respectively

$$\begin{aligned} \varvec{f}=\mathbf {K}^{-1}\varvec{y}-\mathbf {div}(\nu \varvec{\varepsilon }(\varvec{y})-p\mathbf {I}) - \varvec{u},\qquad \varvec{y}_d =\varvec{y}-\mathbf {K}^{-1}\varvec{w}+\mathbf {div}(\nu \varvec{\varepsilon }(\varvec{w})+r\mathbf {I}). \end{aligned}$$

**Fig. 3:**
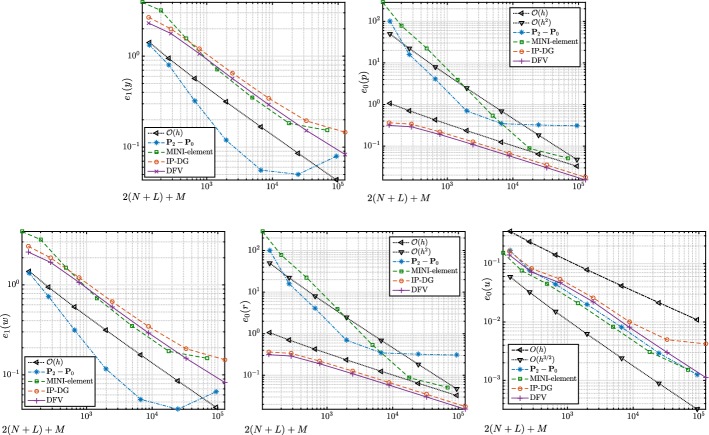
Example 1: comparison between errors generated using a $\mathbb {P}_2-\mathbb {P}_0$, the MINI-element, an interior penalty DG, and a DFV approximation of velocity and pressure in the primal and adjoint problems

A family of nested primal and dual triangulations of $\varOmega$ is generated, on which we compute errors in the $\mathbf {L}^2-$and mesh-dependent norm $\left| \left| \left| \cdot \right| \right| \right| _{1,h}$ for the state and co-state velocity, in the $L^2-$norm for pressures, and in the $\mathbf {L}^2-$norm for the control approximation. Table 1 displays the error history for this first test, where we observe optimal convergence rates for velocity and pressure (only those of the state equation are shown) in their natural norms, along with an *O*(*h*) convergence for the control when approximated by piecewise constant elements, which improves to roughly a $O(h^{3/2})$ rate under piecewise linear approximations. We can also confirm that a maximum of three iterations are needed to reach the stopping criterion that the active sets are equal to those in the previous optimisation step. This indicates a mesh independence of the method in the sense that the number of iterations needed to achieve the stopping criterion is independent of the resolution. In addition we portray in Fig. 2 the obtained approximate solutions at the finest resolution level, where we highlight the active sets with a contour plot on top of the control and state velocities. In all examples herein we employ a BiCGSTAB method with AMG preconditioning to solve the linear systems involved at each step of Algorithm 1. Moreover, the zero-mean pressure condition is applied for both pressure and adjoint pressure using a real Lagrange multiplier approach (which accounts for adding one column and one row to the relevant matrix system).

We also present a basic comparison with other classical methods in terms of accuracy. For instance, we have performed the same test as above but employing model coefficients with jumps, in order to highlight the need for discontinuous approximations. Both fluid viscosity and medium permeability have now a discontinuity of five orders of magnitude at $x_1=0.5$. The tested methods are: a conforming stable $\mathbb {P}_2-\mathbb {P}_0$ and MINI-element pairs for velocity and pressure approximation, a classical interior penalty DG method using the same stabilisation parameters as in (2.4)–(2.5), and the proposed DFV formulation. In all cases we consider a piecewise linear approximation of the control variable.

**Fig. 4:**
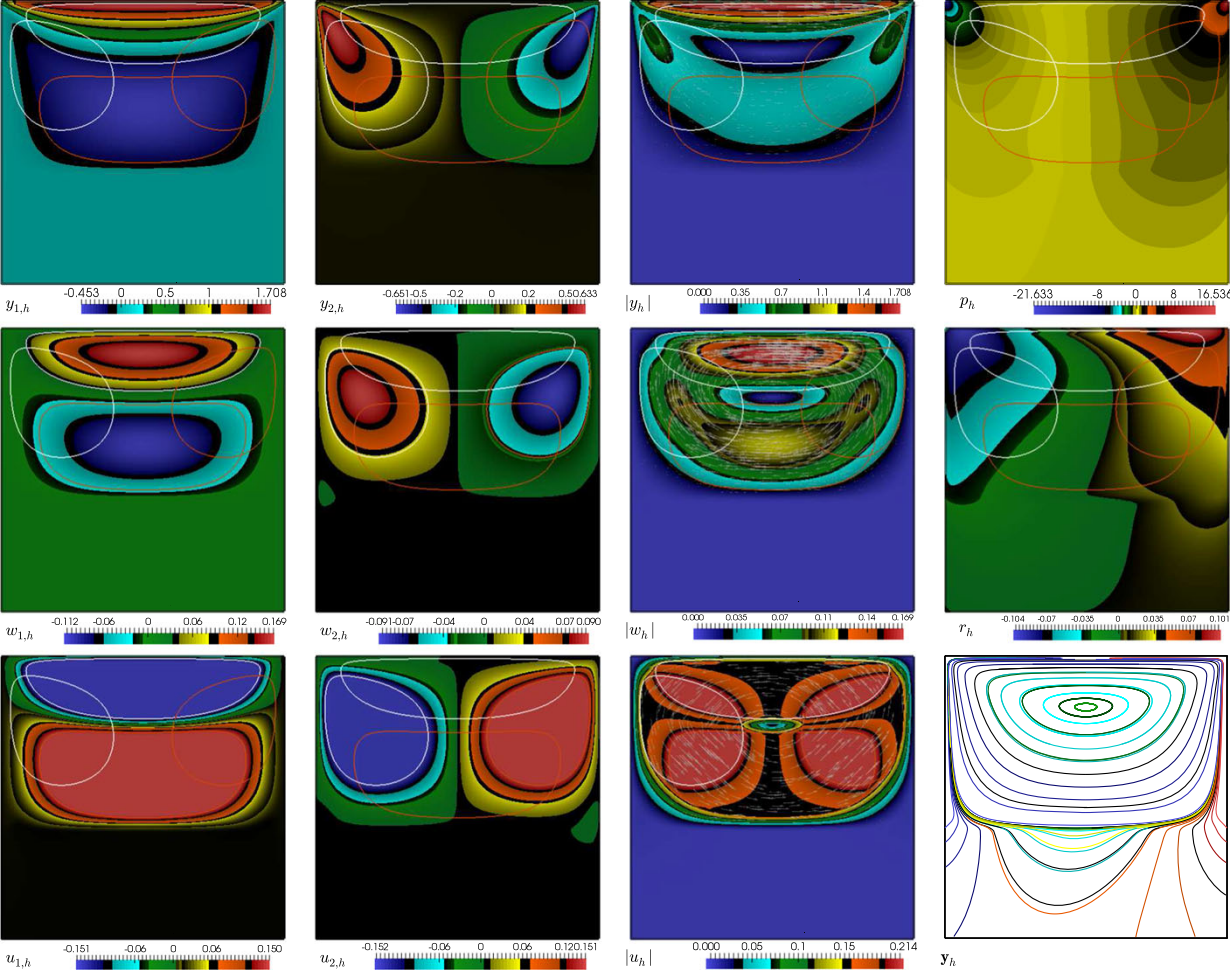
Example 2: DFV approximation of state velocity components and magnitude along with state pressure (top panels), adjoint velocity and pressure (centre row), components and magnitude of the control variable under piecewise constant approximation, and state velocity streamlines (bottom row). Contours of the active sets associated to $a_1=a_2$ (in white curves) and $b_1=b_2$ (red curves) are displayed on each plot (Color figure online)

The results are collected in Fig. 3, where convergence histories (errors for velocity and pressure vs. the number of degrees of freedom DoF$=2(N+L)+M$) associated to the studied discretisations are shown. For all fields, the DFV approximation exhibits a slightly better accuracy than its pure-DG counterpart. This may be explained by the smaller elements used in the dual mesh (but being associated to the same number of DoF). On the other hand, for coarse meshes the conforming approximation $\mathbb {P}_2-\mathbb {P}_0$ outperforms all other methods, but for finer meshes the discontinuous coefficients of the problem imply a badly conditioned system matrix requiring more iterations of the linear solver and eventually the conforming methods lose their optimal convergence. For a fixed number of DoF, the proposed DFV scheme produces smaller errors for the pressure approximation than the other methods. We stress that some recent theoretical comparison results are available for forward Stokes problems (see e.g. [13]), but only in the case of smooth solutions and constant coefficients. If the comparisons are carried out for the case of smooth solutions, then the error estimates in e.g. Theorem 5 are indeed of the same order as their finite element counterpart. However the constants in the estimates are not necessarily the same. As mentioned above, since the dual elements are in principle smaller than the primal ones, the approximate solutions and the corresponding errors generated with discontinuous finite volume schemes are still slightly more localised, implying that the errors themselves are smaller than those produced with methods based on the primal mesh. Complexity, implementation, and CPU times for assembly and solution of the linear systems are, on the other hand, comparable to those associated with classical finite elements.

**Table 2:** Example 2: iteration count versus the regularisation parameter for the DFV approximations of the optimal control of the Brinkman problem

$\lambda$	1	0.2	0.04	0.008	0.0016	0.00032	0.000064
it	5	6	7	8	12	19	34

**Fig. 5:**
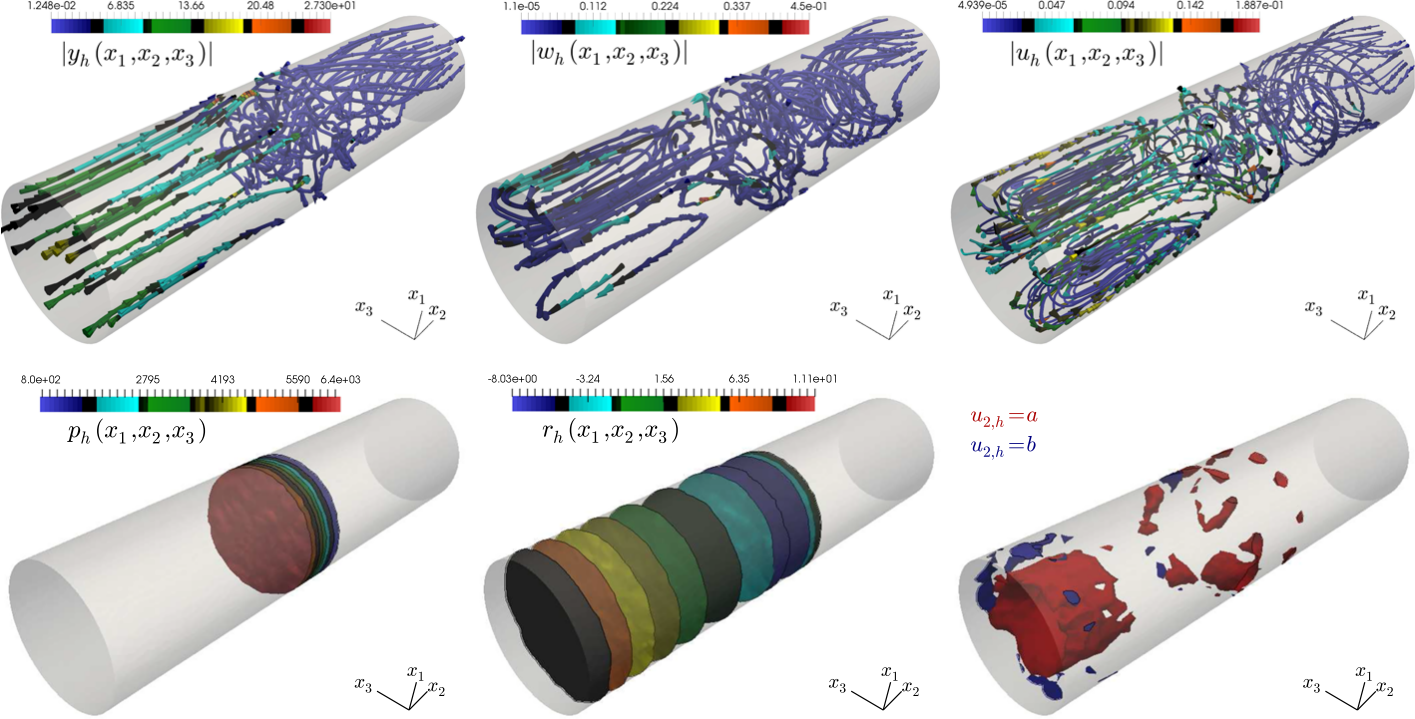
Example 3: streamlines of the DFV approximate state and co-state velocities, along with control field (top row), iso-surfaces of approximate state and co-state pressures together with iso-surfaces of the second component of the control variable associated to $a=a_1=a_2=a_3$ (in red) and $b=b_1=b_2=b_3$ (blue) (bottom panels) (Color figure online)

### Example 2

Our second test focuses on the optimal control problem applied to the well-known lid driven cavity problem. The objective function still corresponds to (1.1), but no analytic exact solution is available. Again the domain consists of the unit square, and the data of the problem are given by a traction boundary condition on the top of the lid, the applied body force, and an observed velocity field $\varvec{y}=(1,0)^T$ on the top and zero elsewhere, and $\varvec{f}=\varvec{y}_d=\varvec{0}$ in $\varOmega$. The adjoint problem is subject to homogeneous Dirichlet data. The viscosity is set to $\nu =0.1$, the control weight is now $\lambda =0.2$, the admissible control space is characterised by $a_1=a_2=-0.15$, $b_1=b_2=0.15$, and the permeability exhibits a discontinuity on the line $x_2=0.4$: $\mathbf {K}(\varvec{x})=\frac{\kappa }{\nu }\mathbf {I}$, with $\kappa (\varvec{x})=\{10{,}000$ if $x_2\ge 0.4$; 10 elsewhere in $\varOmega \}$ (see also [2, 3] for the simulation of Brinkman flows with sharp interfaces). The domain is discretised into 20,000 primal triangular elements, and Fig. 4 portrays all fields obtained with our DFV scheme, where the stabilisation parameter is $\alpha _d=10$. From Fig. 4 we observe that the controlled velocity approaches the desired velocity, that is, it goes to zero and the movement of the fluid concentrates in the upper section of the cavity. In addition, we study the influence of the Tikhonov regularisation in the iteration count of the active set algorithm applied to a coarse solve of this test. As in [25], we immediately observe that a larger number of iterations are required for smaller values of $\lambda$ (see Table 2).

### Example 3

Next we turn to the numerical solution of a three-dimensional optimal control problem (see also [34]). The domain is a cylinder with height 4 and radius 1, aligned with the $x_2$ axis. The permeability field is anisotropic $\mathbf {K}=\mathrm{diag}(0.1,10^{-5}\chi _B+0.1\chi _{B^c}, 0.1)$, where *B* is a ball of radius 1/4 located at the centre of the domain. A Poiseuille inflow profile is imposed as state velocity at $x_2=0$: $\varvec{y}=(0,10(1-{x_1}^2-(x_3-1/2)^2),0)^T$, a zero-pressure is considered on $x_2=4$, whereas homogeneous Dirichlet data are enforced on the remainder of $\partial \varOmega$. The viscosity is $\nu =0.005$, the Tikhonov regularisation is $\lambda =1/2$, the desired velocity is zero $\varvec{y}_d=\varvec{0}$, the bounds for the control are $a_j=a=-0.1$ and $b_j=b=0.2$, and a smooth body force is set as in [4]: $\varvec{f}= \mathbf {K}^{-1}(\exp (-x_2x_3)+x_1\exp (-x_2^2), \cos (\pi x_1)\cos (\pi x_3)-x_2\exp (-x_2^2),-x_1x_2x_3-x_3\exp (-x_3^2))^T$. The primal meshes has 78,631 internal tetrahedral elements and 13,593 vertices. We observe that five iterations are required to reach the stopping criterion (4.1). Snapshots of the resulting approximate fields are collected in Fig. 5.
